# Validation of an acute respiratory infection phenotyping algorithm to support robust computerised medical record-based respiratory sentinel surveillance, England, 2023

**DOI:** 10.2807/1560-7917.ES.2024.29.35.2300682

**Published:** 2024-08-29

**Authors:** William H Elson, Gavin Jamie, Rashmi Wimalaratna, Anna Forbes, Meredith Leston, Cecilia Okusi, Rachel Byford, Utkarsh Agrawal, Dan Todkill, Alex J Elliot, Conall Watson, Maria Zambon, Roger Morbey, Jamie Lopez Bernal, FD Richard Hobbs, Simon de Lusignan

**Affiliations:** 1 Nuffield Department of Primary Care Health Sciences, University of Oxford, Oxford, United Kingdom; 2 Renal services, Epsom and St. Helier University Hospitals NHS Trust, London, United Kingdom; 3 Real-time Syndromic Surveillance Team, United Kingdom Health Security Agency, Birmingham, United Kingdom; 4 Immunisation and Vaccine-Preventable Diseases Division, United Kingdom Health Security Agency, London, United Kingdom; 5 Reference Microbiology, United Kingdom Health Security Agency, London, United Kingdom

**Keywords:** Respiratory Tract Infections, Public Health Surveillance, Sentinel Surveillance, Computerised Medical Records, Electronic Health Records, Phenotype

## Abstract

**Introduction:**

Respiratory sentinel surveillance systems leveraging computerised medical records (CMR) use phenotyping algorithms to identify cases of interest, such as acute respiratory infection (ARI). The Oxford-Royal College of General Practitioners Research and Surveillance Centre (RSC) is the English primary care-based sentinel surveillance network.

**Aim:**

This study describes and validates the RSC’s new ARI phenotyping algorithm.

**Methods:**

We developed the phenotyping algorithm using a framework aligned with international interoperability standards. We validated our algorithm by comparing ARI events identified during the 2022/23 influenza season in England through use of both old and new algorithms. We compared clinical codes commonly used for recording ARI.

**Results:**

The new algorithm identified an additional 860,039 cases and excluded 52,258, resulting in a net increase of 807,781 cases (33.84%) of ARI compared to the old algorithm, with totals of 3,194,224 cases versus 2,386,443 cases. Of the 860,039 newly identified cases, the majority (63.7%) were due to identification of symptom codes suggestive of an ARI diagnosis not detected by the old algorithm. The 52,258 cases incorrectly identified by the old algorithm were due to inadvertent identification of chronic, recurrent, non-infectious and other non-ARI disease.

**Conclusion:**

We developed a new ARI phenotyping algorithm that more accurately identifies cases of ARI from the CMR. This will benefit public health by providing more accurate surveillance reports to public health authorities. This new algorithm can serve as a blueprint for other CMR-based surveillance systems wishing to develop similar phenotyping algorithms.

**Key public health message**

**What did you want to address in this study and why?**

Public health authorities increasingly use routinely collected electronic patient records to identify trends in respiratory infections such as coronavirus or influenza. Computer algorithms are needed to identify respiratory infections in the electronic patient record. We wanted to test a new algorithm for identifying cases of acute respiratory infections from electronic patient records of primary care clinics in England.

**What have we learnt from this study?**

Our new algorithm was more accurate at identifying cases of probable acute respiratory infection in electronic patient records. Overall, it identified 807,781 (34%) more cases of respiratory infection than the old algorithm.

**What are the implications of your findings for public health?**

The new algorithm allows better quality and consistent data to be supplied to public health officials. More accurate data means public health authorities can more reliably identify unusual or increased virus activity. We explain the inner workings of our algorithm which allows it to be used by other scientists or public health professionals, and also allows for healthy scrutiny by other experts in the field.

## Introduction

Respiratory sentinel surveillance involves monitoring a representative sample of the population to identify and track respiratory pathogens of epidemic or pandemic potential. In its 2023 Mosaic framework, the World Health Organization (WHO) sets out how global sentinel surveillance should monitor respiratory viruses [1]. Sentinel systems typically measure the rate of clinical indicators such as influenza-like illness (ILI) and acute respiratory infection (ARI) to monitor community disease. Influenza-like illness is an influenza-specific indicator, whereas ARI is a more inclusive concept, capturing a broader range of clinical presentations [2]. For effective sentinel surveillance, indicators must be reported in a timely manner to determine if health systems are at risk of being overwhelmed [3,4].

The use of routinely collected data, held on computerised medical records (CMR), facilitates systematic and automated computation of indicator rates and can increase the timeliness of surveillance reporting [5]. Most CMRs have inbuilt clinical terminologies, such as Systematized Nomenclature of Medicine (SNOMED) – Clinical Terms (CT) or International Classification of Disease (ICD) with which important clinical data, such as diagnoses and symptoms, are coded [6-9].

Computerised medical record-based surveillance systems use algorithms to identify cases of interest, such as ARI, from the medical records [10]. These case detection algorithms typically include a description of the clinical logic behind the algorithm and clinical codelists that map to a relevant case definition [11]. Finally, the clinical logic and codelists are translated into a machine-readable computer programme. The programme is run against the CMR to extract cases of interest. These algorithms are commonly referred to as phenotyping algorithms and codelists are also known as value sets or refsets [11,12].

The Oxford-Royal College of General Practitioners (RCGP) Research and Surveillance Centre (RSC) runs the English primary care sentinel surveillance network and has been undertaking CMR-based research and surveillance for more than 20 years [13,14]. The RSC provides bi-weekly ARI surveillance and reports to the United Kingdom (UK) Health Security Agency (UKHSA). In late 2023, the RSC updated its methodological approach to developing phenotyping algorithms [11]. This new framework uses international standards of interoperability and supports the principals of open science by allowing algorithms to be published in online libraries in a standard format [15].

Recently, the RSC has used this new framework to update its ARI phenotyping algorithm. The motivation for developing this algorithm was to review and improve the accuracy of ARI case identification. We planned to increase cases correctly identified and reduce cases incorrectly identified. Improving our ARI indicator will benefit public health by providing more accurate and valid surveillance reports to our partners at UKHSA. In addition, and in the spirit of open science, we are publishing the algorithm here and opening it to external scrutiny.

This study aimed to describe and validate the new RSC ARI phenotyping algorithm by comparing the new and old algorithms. Specifically, we undertook three analyses: (i) comparing codelists developed for the new algorithm with those of the old algorithm; (ii) comparing ARI cases identified from the CMR by the new algorithm with those identified by the old algorithm; (iii) comparing the estimated weekly rate of ARI by age group and risk group status using the new vs old algorithms.

## Methods

### Study setting

The RSC works in collaboration with the UKHSA and has been collecting primary care surveillance data since 1957 [13]. Currently, the RSC collects data from the CMR of more than 18 million patients based at more than 1,800 RSC member primary care practices and covers just under a third of the population of England. Coded clinical events are recorded by clinicians during patient encounters and administrators in primary care using SNOMED codes [9]. The RSC receives date-stamped SNOMED-recorded events from all its registered practices.

In addition to monitoring key respiratory indicators, the RSC also undertakes virological sampling of a subset of cases allowing assignment of virological diagnoses [13]. Surveillance reports at the RSC are derived from the primary care data and linked to virological sampling results data. The RSC’s primary care data are available in near real-time, arriving with a lag of only 2–4 days.

### Updated approach to phenotyping algorithms

Health Level 7’s (HL7) Fast Healthcare Interoperability Resources (FHIR) is a widely adopted international standard for exchange of healthcare information [16]. The FHIR can be used in conjunction with Clinical Quality Language (CQL), a healthcare-specific programming language, for sharing phenotyping algorithms in a human- and machine-readable format [17]. We have adopted the Phenotype Execution and Modelling Architecture (PhEMA) approach to phenotyping algorithm development [18]. The PhEMA is a collaborative framework developed by a number of institutions in the United States designed to facilitate the development of CMR-based phenotyping algorithms. It incorporates elements from the FHIR framework and CQL. A description of how the RSC uses this new methodology can be found in Jamie et al. [11].

### Clinical logic

Previously, the RSC monitored ARI through a number of specific surveillance indicators, principally ILI and upper/lower respiratory tract infections (URTI and LRTI). We did not have an overall ARI indicator. For comparison, in this study, the old ARI codelist was defined as a combination of the old ILI, URTI and LRTI codelists.

**Figure 1:**
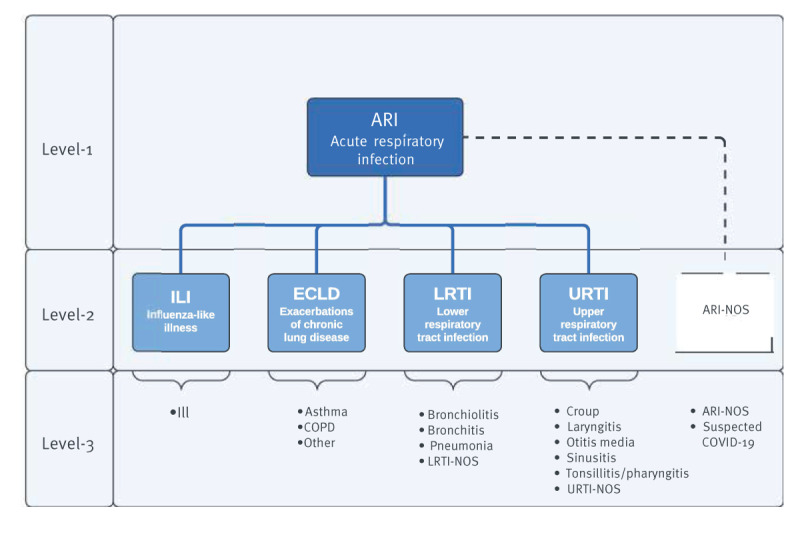
The 3-level hierarchical model of the acute respiratory infection clinical logic

COPD: chronic obstructive pulmonary disease; NOS: not otherwise specified.

We developed our new clinical logic through discussion with clinical, public health and informatics experts in our research group. The clinical logic was designed to capture the range of possible presentations of ARI. We took a practical view that ARI is a hierarchical indicator, with ARI at the top of the hierarchy (level 1), and there being several child (level 2) and grandchild (level 3) indicators. To cover the range of possible infections we included four level 2 indicators in our model: ILI, exacerbation of chronic lung disease (ECLD), LRTI and URTI (Figure 1). We used the 2018 European Union (EU) ARI case definition [19]. We used the RSC case definition for ILI and developed case definitions for ECLD, LRTI and URTI through expert consensus within our group; these definitions are appended in the Supplement, part S1.

The ARI-NOS indicator includes codes not specific enough be included in another level 2 or 3 indicator.

For each of these level 2 indicators, we identified relevant level-3 indicators of which there were 16 in total. Consistent with our previous approach, cases of ARI were defined when ARI events were recorded more than 28 days from the previous recorded event to reduce the chance of duplicate case counting. The CQL files show the machine-readable and human-readable logic of our algorithm; the script is appended in the Supplement, part S2.

The level 1 and 2 indicators represent our main surveillance indicators. Influenza may infect any part of the respiratory tract, therefore ILI could technically be regarded as an URTI or a LRTI [20]. We made the decision to separate ILI into its own level 2 condition to reduce the complexity of the model. This is a practical modification of our clinical logic. In addition, we created a separate level 2 group ‘ARI not otherwise specified (NOS)’ that included two level 3 concepts, a clinical concept where ARI is coded but there is no specific information that allows us to assign it to a level 2 or 3 indicator, and a suspected COVID-19 concept. We produced a flow sheet for clinicians to use with suggested clinical terms which we make available in the Supplement, part S3.

### Codelist development

Like many clinical terminologies, SNOMED codes form a hierarchy. However, a key difference between SNOMED and other terminologies is that SNOMED codes are polyhierarchical where any individual code may have multiple parent codes. This adds complexity but also allows more flexible rules to be developed for selecting codes. SNOMED CT’s Expression Constraint Language (ECL) is a formal language used to define rules for selecting appropriate SNOMED codes, to create dynamic, rule-bound codelists [21]. The new framework takes a rule-based approach and fully leverages the SNOMED polyhierarchy through use of ECL. We used our in-house ‘SNOMED helper tool’ to develop ECL rule-based SNOMED codelists for each level 3 indicator. An example ECL script is available in the Supplement, part S4 and all resultant codelists are provided in Supplement part S5. A total of 16 individual codelists were developed: one for ILI, three for ECLD, four for LRTI, six for URTI and two under the level-2 ‘ARI-NOS’ group. Acute respiratory infection can be defined as a composite of all 16 level-3 codelists. Codelists at levels 1 and 2 were inferred through combinations of level 3 codelists. A more detailed description of how codelists were defined using ECL is available in the Supplement, part S4.

### Algorithm validation

#### Part 1: Codelist comparison

Initially, we compared the old and new codelists. We compared all new level 1 codes (derived by combining all 16 level 3 codelists) with the combination of the old extensional codelists for ILI, LRTI and URTI. To compare these codelists, we performed a set analysis to establish the number of codes present in each codelist and the number of intersecting codes.

#### Part 2: Acute respiratory infection case comparison

We used the old and new algorithms to extract ARI cases from the RSC CMR for the 2022/23 surveillance season, starting from International Organization for Standardization (ISO) week 39 in 2022 and finishing in ISO week 38 in 2023. We compared codes from the old and new algorithms that are responsible for identifying most cases of ARI from the CMR. We also looked at cases identified by the old algorithm that are no longer identified by the new algorithm and codes identified by the new algorithm but not previously identified by the old algorithm.

#### Part 3: Acute respiratory infection weekly rate comparison

We calculated the overall rate of ARI and the rate by age band and risk group as cases per 100,000 by for the surveillance year 2022/23. We used three age bands: 0–17 years, 18–69 years and 70 years and older. Risk groups were defined based on those published in the UK Immunisation Against Infectious Disease Book [22]. We calculated the weekly ARI and level 2 indicator rates using the new algorithm, comparing with the old indicators, and presented these as time series plots of level-2 indicators. No comparison was made between the new ECLD or ARI-NOS indicator as no equivalent existed previously. The data extracted for the analysis for part 3 were not identical to those used for part 2. This is because we did not always have reliable denominator data for every primary care practice. Data quality checks thus eliminated cases from some practices for which a denominator could not be reliably calculated.

### Data analysis

All data required for this analysis were stored in the secure RSC servers within several Structure Query Language (SQL) databases. Further information about the security infrastructure and procedures of the RSC can be seen in the Supplement, part S6. We used an instance of R statistical software (R Core Team, 2023, version 4.3.1) housed within the secure server for all analysis [23]. Only anonymous data, such as aggregated results or summary figures, can be extracted from the secure server.

## Results

### Part 1: Codelist analysis

The old ARI codelist contained 821 unique SNOMED codes compared with 544 in the new codelists, representing a 33.7% (n = 277) reduction in the total number of codes (Table 1). There were 417 codes that appeared in both the old and new codelists, 404 that appeared only in the old and 127 that appeared only in the new codelist; a graphical representation of this is available in the Supplement, part S7. With the new algorithm, 304 of the 544 SNOMED codes were used to record ARI cases. Of these, 25 codes were responsible for identifying 90.5% of all events; therefore, 279 codes were responsible for identifying the remaining cases. For the old algorithm, 346 of the 821 codes were used to record cases. Of these, 16 codes were responsible for 90.6% of all events and 330 for the remaining cases.

**Table 1:** Number of codes within level 1 and level 2 codelists and the corresponding number of acute respiratory infection cases identified during the influenza season, England, 2022/23

Level	Codelist	Codelist size		ARI cases		
		Old	New	Old	New	% Change
Level 1	ARI	821	544	2,386,443	3,194,224	33.8
Level 2	URTI	448	206	1,647,236	1,862,191	13.0
	LRTI	377	243	766,707	987,203	28.7
	ARI-NOS	NA	14	NA	219,310	NA
	ECLD	NA	49	NA	141,482	NA
	ILI	43	49	47,815	47,812	0.0

ARI: acute respiratory infection; ARI-NOS: acute respiratory infection-not otherwise specified; ECLD: exacerbations of chronic lung disease; ILI: influenza-like illness; LRTI: lower respiratory tract infection; NA: no old codelist available for comparison; URTI: upper respiratory tract infection.

As a small number of ambiguous codes may appear in more than one level 2 codelist, the number of codes in level 2 codelists does not sum to the total number in the level 1 codelists.

### Part 2: Acute respiratory infection case analysis

Old and new cases of ARI were derived from the same study population. The new algorithm identified 3,194,224 ARI cases compared with the 2,386,443 cases identified by the old algorithm (Table 1). This represents a 33.8% (n = 807,781) increase in the number of cases detected. Of the cases identified using the old algorithm, 52,258 (2.2%) were no longer identified by the new algorithm. Cases of ARI detected by the new algorithm were most commonly URTIs (58.3%), followed by LRTIs (30.9%), ARI-NOS (6.9%), ECLD (4.4%) and finally ILI (1.5%).

**Figure 2:**
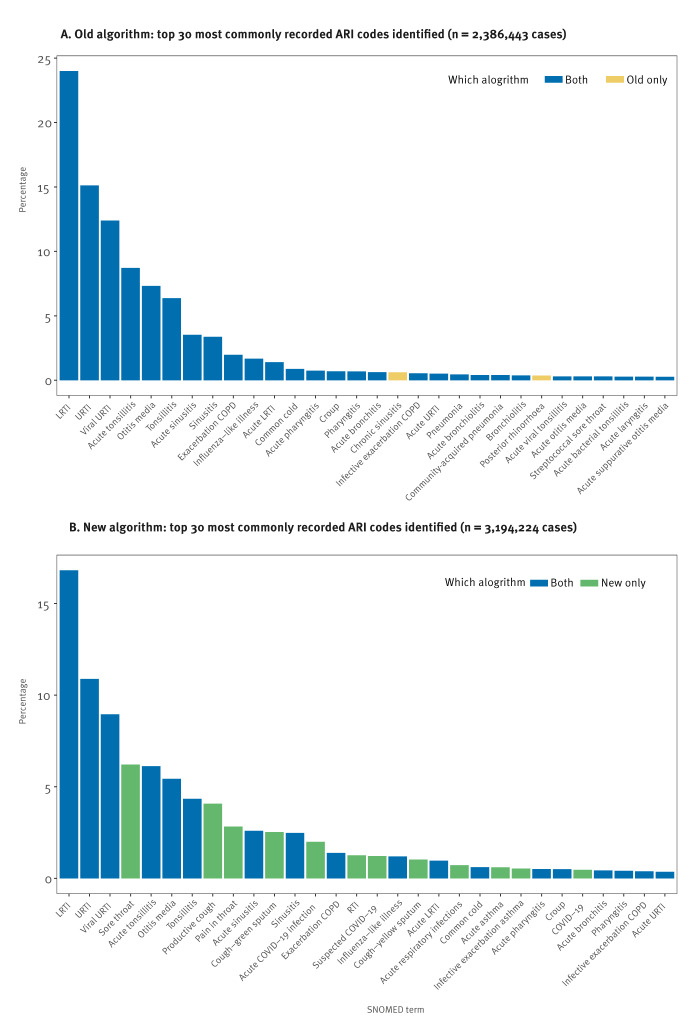
SNOMED code frequency for cases of acute respiratory infection detected by the old and new algorithm, England, 2022/23

ARI: acute respiratory infection; COPD: chronic obstructive pulmonary disease; LRTI: lower respiratory tract infection; RTI: respiratory tract infection; SNOMED: systematised nomenclature of medicine; URTI: upper respiratory tract infection.

The new algorithm identified 28.7% more cases of LRTI, 13.0% more cases of URTI, and an almost identical number of cases of ILI compared with the old algorithm. The three most commonly recorded codes using both the new and old algorithm were ‘Lower respiratory tract infection’, ‘Upper respiratory infection’ and ‘Viral upper respiratory tract infection’ (Figure 2).

The code descriptions have been shortened for formatting purposes.

**Figure 3:**
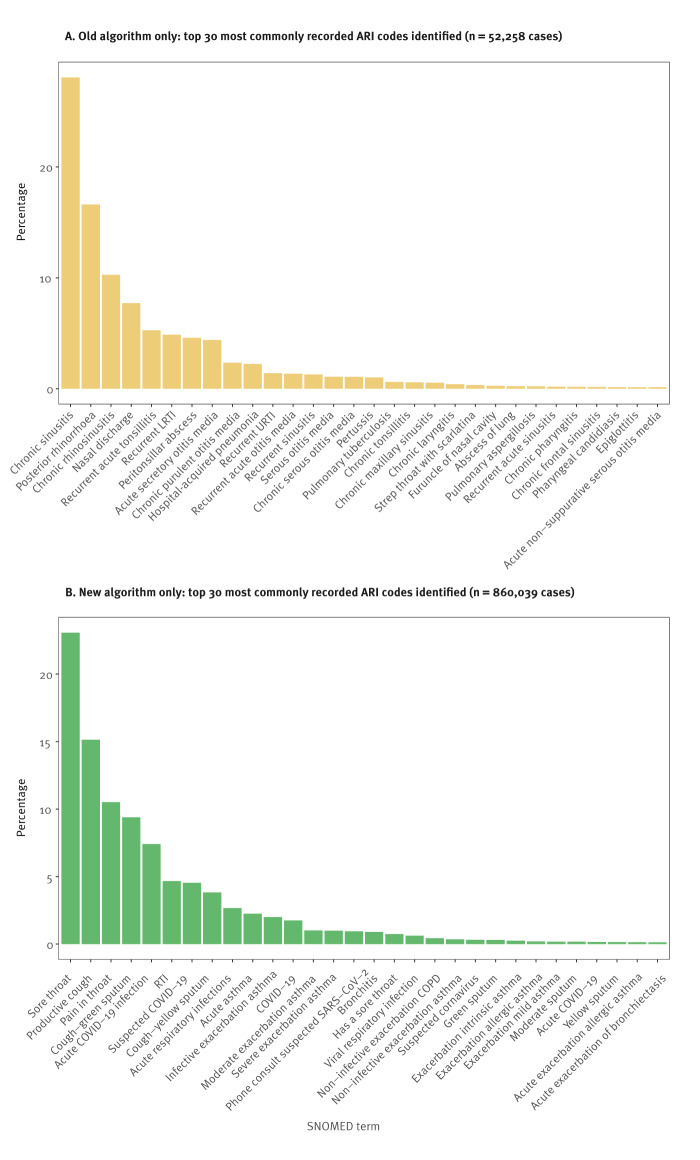
SNOMED code frequency for cases of acute respiratory infection *only* identified using the old algorithm or new algorithm, England, 2022/23

ARI: acute respiratory infection; COPD: chronic obstructive pulmonary disease; LRTI: lower respiratory tract infection; RTI: respiratory tract infection; SARS-CoV-2: Severe acute respiratory syndrome coronavirus 2; SNOMED: systematised nomenclature of medicine; URTI: upper respiratory tract infection.

Thirteen codes were responsible for 90.4% of the 52,258 ARI cases no longer detected by the new algorithm (Figure 3). The reasons these cases were no longer included were varied. Of the 52,258 cases now excluded, 22,862 (43.7%) represented chronic conditions, 15,695 (30.0%) were non-infective conditions and 7,515 (14.4%) represented recurrent disease. Thirteen codes were responsible for 91.1% of the 860,039 cases of ARI now included by the new algorithm but not included with the old algorithm (Figure 3). Of the 860,039 cases now included, 547,550 (63.7%) were symptomatic codes that very probably represented ARI cases, 199,299 (23.2%) represented ARI-NOS cases and 71,464 (8.3%) were ECLD cases.

The code descriptions have been shortened for formatting purposes.

#### Part 3: Acute respiratory infection weekly rate analysis

**Figure 4:**
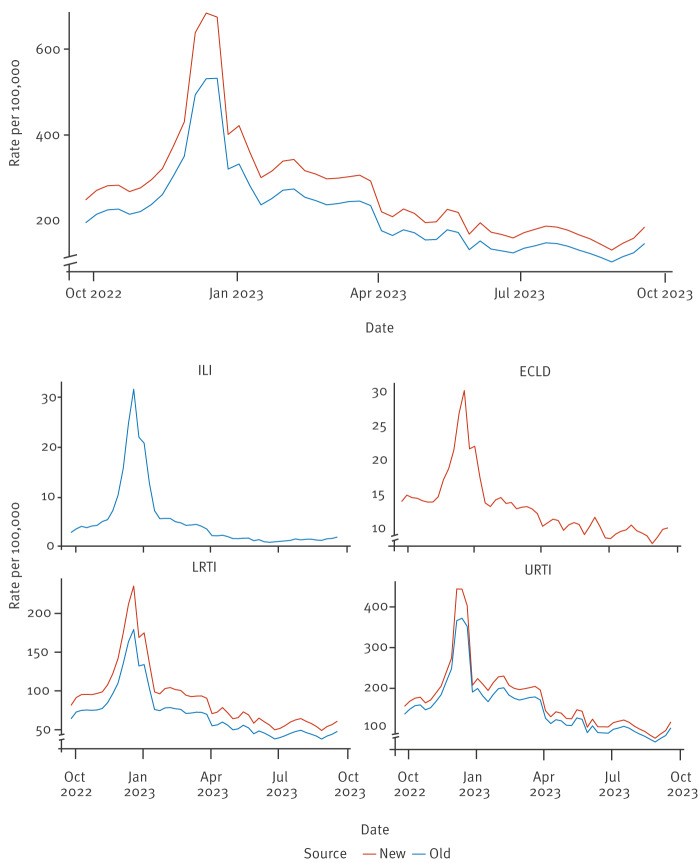
Weekly acute respiratory infection indicator rates old vs new algorithm, England, 2022/23 (old n = 1,965,341, new n = 2,478,473)

ECLD: exacerbations of chronic lung disease, ILI: influenza-like illness; LRTI: lower respiratory tract infection, URTI: upper respiratory tract infection.

When looking at weekly ARI rates per 100,000 of the population, we excluded practices that did not provide reliable denominator data, thus case numbers were less than shown in Table 1. The new ARI algorithm identified 2,478,473 cases from practices with reliable denominator data during the study period compared with 1,965,341 cases with the old, representing an additional 513,132 cases (Table 2). The median ARI weekly rate increased from 205.6 to 258.9 per 100,000 population, representing a 25.9% increase with the new algorithm. While rates of indicators increased when using the new algorithm, trends over the course of the 2022/23 season remained the same (Figure 4). There was no meaningful change in the rates of ILI across all age bands and risk group categories (Table 2). However, rates increased across all age bands and risk groups categories for ARI as a whole, LRTI and URTI; we append the detailed numbers in the Supplement, part S8.

**Table 2:** Median weekly rate of respiratory infection per 100,000 of the population for level-1 and level-2 indicators using new and old algorithms, England, 2022/23

Level	Indicator	ARI cases		Weekly rate/100,000		
		Old	New	Old	New	% Change
Level 1	ARI	1,965,341	2,478,473	205.6	258.9	25.9
Level 2	URTI	1,357,932	1,556,881	140.7	159.0	13.0
	LRTI	639,062	827,064	67.4	86.4	28.1
	ECLD	NA	119,036	NA	12.5	NA
	ILI	46,529	46,526	3.1	3.1	0.0

ARI: acute respiratory infection; ECLD: exacerbations of chronic lung disease; ILI: influenza-like illness; LRTI: lower respiratory tract infection; NA: no old indicator available for comparison; URTI: upper respiratory tract infection.

Note that ECLD is a new indicator and does not have a suitable comparator. The rate is based on a mean weekly denominator of 17,433,074 subjects. As a small number of ambiguous codes may appear in more than one level 2 codelist, the number of codes in level 2 codelists does not sum to the total number in the level 1 codelists (n = 513,132). The rate of ARI-NOS was not available as these data are reported prospectively and ARI-NOS rate is not currently calculated separately.

Trends of weekly indicator rates per 100,000 of the population from the old and new indicators. Note, no new indicator is seen for ILI as the rates are nearly identical. No old indicator is seen for ECLD as there was no old comparator. The y-axes are on different scales to allow adequate comparison of old and new trend lines.

## Discussion

We developed a new digital phenotyping algorithm for identifying cases of ARI from the CMR to support the RSC’s respiratory sentinel surveillance programme. Use of this algorithm has improved the overall accuracy of ARI case detection. To develop this algorithm, we used the RSC’s newly adopted framework built around international standards of interoperability. Publication of the algorithm definitions and codelists upholds the principles of open science and allows use of these methods as a blueprint for others in the field of CMR-based surveillance [24].

Our new algorithm improved the accuracy of ARI case detection by increasing correctly identified cases by 33.8% and reducing incorrectly identified cases by 2.2%. Overall, this resulted in a 25.9% increase in the estimated median weekly rate of ARI for the influenza surveillance year 2022/23. This included a substantial increase in the rate of URTI and LRTI. The increase in detected cases was largely driven by the inclusion of new symptom codes and the newly included ARI-NOS/ECLD codelists, whereas the reduction in incorrectly identified cases was due to the removal of codes representing mainly chronic disease, recurrent disease and complications of ARI. Calculation of new 5-year averages for ARI and level 2 indicators using retrospective data allows changes in rates to be correctly interpreted despite changes in codelists.

The improved accuracy of ARI case detection has important public health implications. More accurate case identification enhances the robustness and validity of surveillance reports for our UKHSA partners. The estimated rates we now report are more likely to be reflective of the true rate of ARI in primary care. The inclusion of new codes increases the sensitivity of the surveillance system to detect low levels of disease. This is of value when aggregating the rate across a range of variables such as age, risk group, location and vaccination status. This aids identification of groups disproportionately affected by ARI. Although the reduction in incorrectly identified cases was small (2.2%), it represents an important incremental improvement in the quality of the indicator. The system is no longer susceptible to false-positive surges of ARI caused by increased recording of these inaccurate codes.

The case counts and rate of ILI overall and by age band and risk group did not change. The similarity in ILI case numbers occurred as the new and old ILI codelists were very similar. This points to the fact that our old ILI indicator was well maintained and is reflective of its historical public health importance. However, non-influenza viruses such as severe acute respiratory syndrome coronavirus 2 (SARS-CoV-2) and respiratory syncytial virus (RSV) may present in a clinically heterogeneous manner. Having robust ARI and ILI indicators supports integrated surveillance of a range of respiratory viruses including SARS-CoV-2 and RSV [25]. This also makes the system more robust to the emergence of new respiratory pathogens presenting with varied clinical features.

Using the RSC’s new framework for developing phenotyping algorithms was beneficial for two main reasons. Firstly, the use of FHIR-based international standards supports the future sustainability and interoperability of our systems. This feature will be important as the RSC evolves its secure links to other healthcare-based data systems. Secondly, adopting human- and machine-readable languages (CQL and SNOMED’s ECL) facilitates sharing of interpretable algorithm definitions [18]. While ECL is fully integrated into our system, we are still working towards full integration of CQL. Finally, the standards used here could be applied to any CMR-based surveillance system internationally. Although not all systems use SNOMED, the principles could be applied broadly. The main challenge is that the technical bar to developing such systems is high.

The WHO Mosaic Framework describes the surveillance activities that should be undertaken nationally to support comprehensive respiratory surveillance [1]. The RSC plays a growing role in all three Mosaic domains: detection, characterisation and intervention evaluation. The new algorithm will support disease detection through its enhanced accuracy [26]. With a clearly defined ARI population we will be better placed to characterise cases, for example, associated symptoms, signs and severity. We are already planning the development of a severe-ARI (SARI) indicator to support characterisation of disease severity. The RSC already undertakes intervention evaluation through well-established vaccine effectiveness studies [27].

We have highlighted a number of strengths of this work, but also acknowledge a number of limitations. Firstly, the CMR is primarily a tool to support clinical management of patients. The system relies on high-quality coding from primary care practitioners and therefore, as with all CMR-based surveillance data, quality is a challenge. However, we have provided educational material to primary health care centres in the RSC network to facilitate effective ARI coding, which is also appended here in the Supplement, part S3. Secondly, the inclusion of ECLD could identify non-infective exacerbations. However, many ECLDs are infective and probably represent an unrecognised cohort in our previous surveillance reports. The data presented show that the ECLD rate peaks in winter, suggesting many of these exacerbations are likely to be infective. Also, we recognise that many cases of URTI and LRTI probably occur in individuals with underlying chronic lung disease. At present, the ECLD codelist includes codes that mention an exacerbation and a chronic disease in a single code, for example ‘exacerbation of asthma’, and we do not assign patients to a risk group (such as chronic lung disease) prospectively for our surveillance report due to the computational expense. Despite this, when looking at the overall ARI indicator, we can say we are detecting previously unidentified cases. We plan to review the inclusion of ECLD at the end of the next season. Finally, although virological samples are taken in a proportion of patients with ARI, we have no clear reference standard for what truly represents a case of ARI and therefore calculating sensitivity and specificity is difficult. Comparing the old with the new algorithm has helped to us overcome this challenge. In the future, we plan to undertake an analysis linked to the virology data.

## Conclusion

The development of our new phenotyping algorithm represents an advancement in the RSC's respiratory surveillance. This work has increased the accuracy of our public health reporting, which increases the RSC’s ability to meet key respiratory surveillance standards set out by WHO. Moving forward, our work emphasises the need for continued research to refine ARI coding in primary care, which will improve data quality and aid in the characterisation of disease severity, ultimately contributing to more effective public health responses. Furthermore, sharing of our algorithm specification supports collaborative global surveillance efforts and disseminates innovation that can further automated CMR-based surveillance.

## Supplementary Data

1139000 Supplement

## References

[1] World Health Organization (WHO). “Crafting the Mosaic” a framework for resilient surveillance for respiratory virus and epidemic and pandemic potential. Geneva: WHO; 2023. Available from: https://www.who.int/publications/i/item/9789240070288

[2] European Centre for Disease Prevention and Control (ECDC). Operational considerations for respiratory virus surveillance in Europe. Stockholm: ECDC; 2022. Available from: https://www.ecdc.europa.eu/en/publications-data/operational-considerations-respiratory-virus-surveillance-europe

[3] Jajosky RA Groseclose SL. Evaluation of reporting timeliness of public health surveillance systems for infectious diseases. BMC Public Health. 2004;4(1):29. 10.1186/1471-2458-4-29 15274746 PMC509250

[4] Dailey L Watkins RE Plant AJ. Timeliness of data sources used for influenza surveillance. J Am Med Inform Assoc. 2007;14(5):626-31. 10.1197/jamia.M2328 17600101 PMC1975801

[5] Birkhead GS Klompas M Shah NR. Uses of electronic health records for public health surveillance to advance public health. Annu Rev Public Health. 2015;36(1):345-59. 10.1146/annurev-publhealth-031914-122747 25581157

[6] World Health Organization (WHO). International Statistical Classification of Diseases and Related Health Problems (ICD). Geneva: WHO. [Accessed: 21 Nov 2023]. Available from: https://www.who.int/standards/classifications/classification-of-diseases

[7] NHS England. SNOMED CT - NHS Digital. [Accessed: 24 Mar 2023]. Available from: https://digital.nhs.uk/services/terminology-and-classifications/snomed-ct

[8] Willett DL Kannan V Chu L Buchanan JR Velasco FT Clark JD SNOMED CT concept hierarchies for sharing definitions of clinical conditions using electronic health record data. Appl Clin Inform. 2018;9(3):667-82. 10.1055/s-0038-1668090 30157499 PMC6115233

[9] International Health Terminology Standards Development Organisation (SNOMED International). SNOMED CT: Systematized Nomenclature of Medicine - Clinical Terms. London: SNOMED International. [Accessed: 2 Aug 2024]. Available from: https://www.snomed.org

[10] Brandt PS Kho A Luo Y Pacheco JA Walunas TL Hakonarson H Characterizing variability of electronic health record-driven phenotype definitions. J Am Med Inform Assoc. 2023;30(3):427-37. 10.1093/jamia/ocac235 36474423 PMC9933077

[11] Jamie G Elson W Kar D Wimalaratna R Hoang U Meza-Torres B Phenotype execution and modeling architecture to support disease surveillance and real-world evidence studies: English sentinel network evaluation. JAMIA Open. 2024;7(2):ooae034. 10.1093/jamiaopen/ooae034 38737141 PMC11087727

[12] Giménez-Solano VM Maldonado JA Boscá D Salas-García S Robles M. Definition and validation of SNOMED CT subsets using the expression constraint language. J Biomed Inform. 2021;117:103747. 10.1016/j.jbi.2021.103747 33753269

[13] Leston M Elson WH Watson C Lakhani A Aspden C Bankhead CR Representativeness, vaccination uptake, and COVID-19 clinical outcomes 2020-2021 in the UK Oxford-Royal College of General Practitioners Research and Surveillance Network: cohort profile summary. JMIR Public Health Surveill. 2022;8(12):e39141. 10.2196/39141 36534462 PMC9770023

[14] Deckers JGM Paget WJ Schellevis FG Fleming DM. European primary care surveillance networks: their structure and operation. Fam Pract. 2006;23(2):151-8. 10.1093/fampra/cmi118 16464870

[15] Wilkinson MD Dumontier M Aalbersberg IJ Appleton G Axton M Baak A The FAIR Guiding Principles for scientific data management and stewardship. Sci Data. 2016;3(1):160018. 10.1038/sdata.2016.18 26978244 PMC4792175

[16] Health Level Seven (HL7) International. Fast Health Interoperability Resources (FHIR) Release 5. v5.0.0. Ann Arbor: HL7 International. [Accessed: 21 Nov 2023]. Available from: https://www.hl7.org/fhir

[17] Health Level Seven (HL7) International. Clinical quality language (CQL). Ann Arbor: HL7 International. [Accessed: 21 Nov 2023]. Available from: https://cql.hl7.org

[18] Brandt PS Pacheco JA Adekkanattu P Sholle ET Abedian S Stone DJ Design and validation of a FHIR-based EHR-driven phenotyping toolbox. J Am Med Inform Assoc. 2022;29(9):1449-60. 10.1093/jamia/ocac063 35799370 PMC9382394

[19] European Commission (EC). Commission Implementing Decision 2018/945 of 22 June 2018 on the communicable diseases and related special health issues to be covered by epidemiological surveillance as well as relevant case definitions. Official Journal of the European Union. 2018;61: 24. Available: https://eur-lex.europa.eu/legal-content/EN/TXT/PDF/?uri=OJ:L:2018:170:FULL

[20] Boktor SW, Hafner JW. Influenza. In: StatPearls. Treasure Island (FL): StatPearls Publishing; 2023. Available from: https://www.ncbi.nlm.nih.gov/books/NBK459363

[21] International Health Terminology Standards Development Organisation (SNOMED International). Expression constraint language - specification and guide. London: SNOMED International. [Accessed: 21 Nov 2023]. Available from: https://confluence.ihtsdotools.org/display/DOCECL

[22] UK Health Security Agency (UKHSA). Immunisation of individuals with underlying medical conditions: the green book, chapter 7. London: UKHSA. [Accessed: 15 May 2024]. Available from: https://www.gov.uk/government/publications/immunisation-of-individuals-with-underlying-medical-conditions-the-green-book-chapter-7

[23] R Core Team. R: A language and environment for statistical computing. Vienna: R Foundation for Statistical Computing; 2023. Available from: https://www.R-project.org

[24] David R Rybina A Burel JM Heriche JK Audergon P Boiten JW "Be sustainable": EOSC-Life recommendations for implementation of FAIR principles in life science data handling. EMBO J. 2023;42(23):e115008. 10.15252/embj.2023115008 37964598 PMC10690449

[25] Elson W Zambon M de Lusignan S. Integrated respiratory surveillance after the COVID-19 pandemic. Lancet. 2022;400(10367):1924-5. 10.1016/S0140-6736(22)02325-X 36463902 PMC9714972

[26] Gu X Watson C Agrawal U Whitaker H Elson WH Anand S Postpandemic Sentinel Surveillance of Respiratory Diseases in the Context of the World Health Organization Mosaic Framework: Protocol for a Development and Evaluation Study Involving the English Primary Care Network 2023-2024. JMIR Public Health Surveill. 2024;10:e52047. 10.2196/52047 38569175 PMC11024753

[27] Whitaker HJ Tsang RSM Byford R Andrews NJ Sherlock J Sebastian Pillai P Pfizer-BioNTech and Oxford AstraZeneca COVID-19 vaccine effectiveness and immune response amongst individuals in clinical risk groups. J Infect. 2022;84(5):675-83. 10.1016/j.jinf.2021.12.044 34990709 PMC8720678

[28] Department of Health. The Health Service (Control of Patient Information) Regulations 2002. 2002/1438. London: Department on Health; 2002. Available from: https://www.legislation.gov.uk/uksi/2002/1438/regulation/3

