# Supplementary material for: Validation of an acute respiratory infection phenotyping algorithm to support robust computerised medical record-based respiratory sentinel surveillance, England, 2023
Source: Euro Surveill. 2024 Aug 29;29(35):2300682. doi: 10.2807/1560-7917.ES.2024.29.35.2300682 (PMC11484335; doi:10.2807/1560-7917.ES.2024.29.35.2300682)
Supplement: Supplement [file 23-00682_ELSON_Supplement.pdf]

DISCLAIMER: "This supplementary material is hosted by *Eurosurveillance* as supporting information alongside the article: **Validation of an acute respiratory infection (ARI) phenotyping algorithm to support robust computerised medical record-based respiratory sentinel surveillance: English sentinel network approach,** on behalf of the authors, who remain responsible for the accuracy and appropriateness of the content. The same standards for ethics, copyright, attributions and permissions as for the article apply. Supplements are not edited by *Eurosurveillance* and the journal is not responsible for the maintenance of any links or email addresses provided therein."

SUPPLEMENT S1: CASE DEFINITIONS

ACUTE RESPIRATORY INFECTION (ARI)

Acute respiratory infection (ARI) European Union case definition, 2018 [1]**.**

- *Sudden onset of symptoms*

AND

- *At least one of the following four respiratory symptoms*
  (1) Cough
  (2) Sore throat
  (3) Shortness of breath
  (4) Coryza

AND

- *A clinician's judgement that the illness is due to an infection*

INFLUENZA-LIKE ILLNESS (ILI)

We used the Royal College of General Practitioners Research and Surveillance (RCGP-RSC) ILI case definition [2].

"An acute respiratory illness with a temperature measured as, reported as, or plausibly ≥38 °C and cough, with onset within the past 10 days. ILI cases should not have another more plausible diagnosis. ILI cases have a sudden onset, and there are often symptoms suggestive of systemic upset—myalgia, fatigue, malaise, headache, etc. RCGP RSC stresses the notion of symptoms within 10 days of onset to differentiate acute episodes, with swabs only wanted within 7 days of onset."

UPPER RESPIRATORY TRACT INFECTION (URTI)

We created case definitions for URTI based consensus within our team of public health, clinical and informatics experts.

An acute infection of the respiratory tract above and including the larynx, encompassing the nasal mucosa, paranasal sinuses, middle ear, pharynx and larynx.

LOWER RESPIRATORY TRACT INFECTION (LRTI)

We created case definitions for LRTI based consensus within our team of public health, clinical and informatics experts.

An acute infection of the respiratory tract below the larynx, including bronchus, bronchioles and alveoli.

EXACERBATIONS OF CHRONIC LUNG DISEASE (ECLD)

We created case definitions for ECLD based consensus within our team of public health, clinical and informatics experts.

A sudden deterioration in respiratory symptoms such as cough, sputum production or breathlessness in an individual with underlying lung disease including chronic obstructive pulmonary disease, asthma and other chronic lung diseases.

SUPPLEMENT S2: ARI CLINICAL QUALITY LOGIC (CQL)

The clinical quality language (CQL) script for the acute respiratory infection (ARI) phenotype definition.

~\Downloads\Composite ARI(1).cql

```
/**
 * Orchid Phenotype
 *
 * Name : All ARI Acute Respiratory Tract Infections
 * ID   : 1
 * Url  : https://orchid.phc.ox.ac.uk/phenotype/138
 */
library "All ARI Acute Respiratory Infections" version '1.0.0'

using FHIR version '4.0.0'

include FHIRHelpers version '4.0.0' called FHIRHelpers


valueset "URTI - NOS": '7212: ARI-UpperRespiratoryTractInfectionNOS'
valueset "Croup": '7204: ARI-Croup'
valueset "Laryngitis": '7197: ARI-Laryngitis'
valueset "Otitis Media": '7210: ARI-OtitisMedia'
valueset "Sinusitis": '7206: ARI-Sinusitis'
valueset "Tonsillitis and Phayngitis": '7208: ARI-TonsillitisPharyngitis'
valueset "LRTI - NOS": '7214: ARI-LowerRespiratoryTractInfectionNOS'
valueset "Bronchiolitis": '7213: ARI-Bronchiolitis'
valueset "Bronchitis": '7211: ARI-Bronchitis'
valueset "Pneumonia": '7205: ARI-Pneumonia'
valueset "ARI - NOS": '7222: ARI-AcuteRespiratoryInfection'
valueset "Asthma Exacerbation": '7215: ARI-AsthmaExacerbation'
valueset "COPD Exacerbation": '7216: ARI-ChronicObstructivePulmonaryDiseaseExacerbation'
valueset "Respiratory Disease Exacerbation": '7221: ARI-RespiratoryDiseaseExacerbations'
valueset "Influenza Like Illness": '7199: ARI-InfluenzaLikeIllness'
valueset "Suspected Covid": '7223: ARI-CovidSuspected'

context Patient

define function "Previous Episodes in 28 days"(
    dt System.DateTime
):
    (Condition: "All Acute Respiratory Tract Infections") C
        where
            dt before PhEMAHelpers."Effective Date"(C)
                and
            difference in days between dt
                and
            PhEMAHelpers."Effective Date"(C) < 28
        return
             PhEMAHelpers."Effective Date"(C)

define function "New ARI Episode":
    (Condition: "All Acute Respiratory Tract Infections") ILI1
        where
            Count(
                "Previous Episodes in 28 day"(PhEMAHelpers."Effective Date"(ILI1))
            ) =0

define "All Acute Respiratory Tract Infections":
    flatten {[Condition: "Croup"],
    [Condition: "Laryngitis"],
```



```
    [Condition: "URTI - NOS"],
    [Condition: "Otitis Media"],
    [Condition: "Sinusitis"],
    [Condition: "Tonsillitis and Phayngitis"],
    [Condition: "LRTI - NOS"],
    [Condition: "Bronchiolitis"],
    [Condition: "Bronchitis"],
    [Condition: "Pneumonia"],
    [Condition: "ARI - NOS"],
    [Condition: "Asthma Exacerbation"],
    [Condition: "COPD Exacerbation"],
    [Condition: "Respiratory Disesae Exacerbation"],
    [Condition: "Influenza Like Illness"],
    [Condition: "Suspected Covid"]}


define "case":
    "New ARI episode"
```

SUPPLEMENT S3: PHYSICIAN FLOW CHART FOR RECORDING ARI

Flow chart provided to RSC member primary care practices to support accurate coding of ARI cases by clinicians. We generally try to discourage coding of ARI-NOS as this allows us to be more specifically assign cases to a level 2 indicator. ARI-NOS is therefore not included in this flow chart.

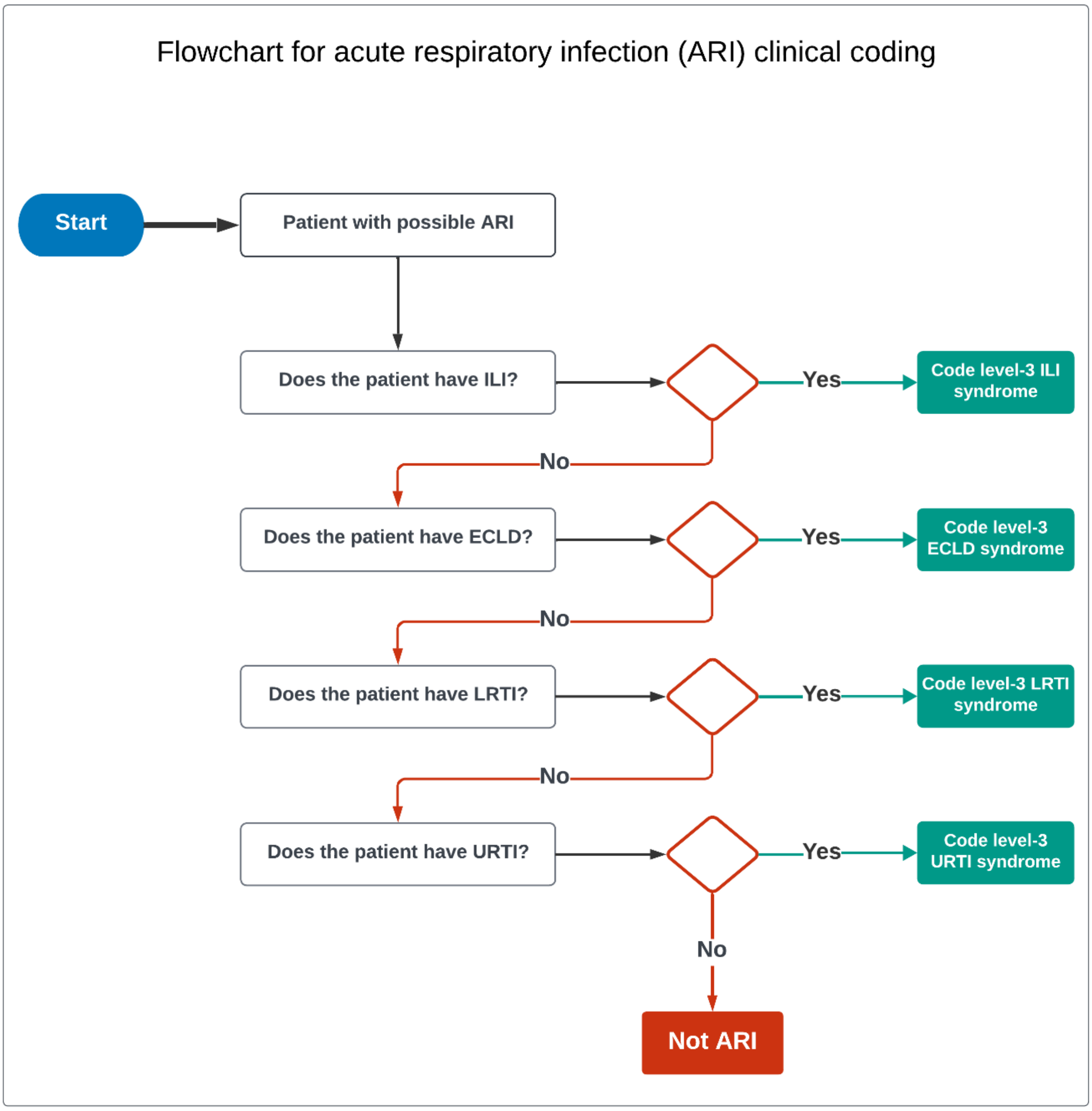

SUPPLEMENT S4: EXPRESSION CONSTRAINT LANGUAGE (ECL)

Example of SNOMED Expression Constraint Language (ECL) for pharyngitis.

Most codelists were defined using the following three steps: **Step 1:** Inclusion of a principal SNOMED supertype(s) and all its descendant codes. For example, we include sinusitis and all is descendant codes, such as frontal sinusitis **Step 2:** We usually did not want to include all descendant codes in the final codelist so rather than pick out individual codes to remove we excluded codes at a higher level in the hierarchy. For example, we excluded a code for chronic disease, which are not relevant to ARI. This has the effect of removing all sinusitis descendant codes that represent instances of chronic sinusitis. **Step 3:** Finally, each codelist needed inclusion or exclusion of specific codes only, allowing for tailored changes to meet the needs of the specific codelist.

```
<< 405737000 | Pharyngitis |          STEP 1
MINUS (
  << 281647001 | Adverse reaction | OR
  << 85828009 | Autoimmune disease | OR
  << 27624003 | Chronic disease | OR
  << 66091009 | Congenital disease | OR
  << 312087002 | Disorder following clinical procedure | OR
  << 414025005 | Disorder of fetus or newborn | OR
  << 17322007 | Parasitic disease | OR                STEP 2
  << 443138004 | Granulomatosis | OR
  << 408678008 | Healthcare associated infectious disease | OR
  << 473010000 | Hypersensitivity condition | OR
  << 41969006 | Idiopathic disease | OR
  << 3218000 | Mycosis | OR
  << 399981008 | Neoplasm and/or hamartoma | OR
  << 441456002 | Polyp | OR
  << 56717001 | Tuberculosis | OR
  << 275488008 | Chronic sore throat |          STEP 3
)
```

SUPPLEMENT S5: CODELISTS

Codelists generated from use of SNOMED ECL for all sixteen new level 3 indicators:

1) ARI-AsthmaExacerbation
2) ARI-ChronicObstructivePulmonaryDiseaseExacerbation
3) ARI-RespiratoryDiseaseExacerbations
4) ARI-InfluenzaLikeIllness
5) ARI-Bronchiolitis
6) ARI-Bronchitis
7) ARI-LowerRespiratoryTractInfectionNOS
8) ARI-Pneumonia
9) ARI-AcuteRespiratoryInfectionNOS
10) ARI-CovidSuspected
11) ARI-Croup
12) ARI-Laryngitis
13) ARI-OtitisMedia
14) ARI-Sinusitis
15) ARI-TonsillitisPharyngitis
16) ARI-UpperRespiratoryTractInfectionNOS

Level 3 indicator	SNOMED ConceptID	SNOMED Preferred term
ARI-Laryngitis	#10809006	Parainfluenza virus laryngotracheitis
ARI-Laryngitis	#13617004	Tracheobronchitis
ARI-Laryngitis	#14969004	Catarrhal laryngitis
ARI-Laryngitis	#195680000	Acute oedematous laryngitis
ARI-Laryngitis	#195681001	Acute ulcerative laryngitis
ARI-Laryngitis	#195682008	Acute catarrhal laryngitis
ARI-Laryngitis	#195683003	Acute phlegmonous laryngitis
ARI-Laryngitis	#195684009	Acute laryngitis caused by Haemophilus influenzae
ARI-Laryngitis	#195685005	Acute pneumococcal laryngitis
ARI-Laryngitis	#195686006	Acute suppurative laryngitis
ARI-Laryngitis	#195709006	Pharyngolaryngitis
ARI-Laryngitis	#195923003	Influenza with laryngitis
ARI-Laryngitis	#232426008	Acute simple laryngitis
ARI-Laryngitis	#232428009	Acute membranous laryngitis
ARI-Laryngitis	#232429001	Acute subglottic laryngitis
ARI-Laryngitis	#233799004	Acute toxic tracheobronchitis
ARI-Laryngitis	#26650005	Acute tracheitis
ARI-Laryngitis	#301824001	Acute viral laryngotracheitis
ARI-Laryngitis	#312400008	Acute infective tracheobronchitis
ARI-Laryngitis	#312423006	Infective laryngitis
ARI-Laryngitis	#32904004	Pneumococcal laryngitis
ARI-Laryngitis	#35301006	Acute tracheobronchitis

ARI-Laryngitis	#37426002	Ulcerative laryngitis
ARI-Laryngitis	#37948003	Acute laryngotracheitis without obstruction
ARI-Laryngitis	#408669002	Acute laryngitis with obstruction
ARI-Laryngitis	#41048006	Haemophilus influenzae laryngitis
ARI-Laryngitis	#441551009	Viral laryngitis
ARI-Laryngitis	#45913009	Laryngitis
ARI-Laryngitis	#50211006	Catarrhal tracheitis
ARI-Laryngitis	#55130001	Laryngotracheitis
ARI-Laryngitis	#55355000	Acute laryngopharyngitis
ARI-Laryngitis	#59967003	Acute laryngotracheitis with obstruction
ARI-Laryngitis	#62994001	Tracheitis
ARI-Laryngitis	#64369009	Acute tracheitis without obstruction
ARI-Laryngitis	#64375000	Acute laryngotracheitis
ARI-Laryngitis	#66011008	Viral tracheitis
ARI-Laryngitis	#6655004	Acute laryngitis
ARI-Laryngitis	#709663002	Supraglottitis
ARI-Laryngitis	#82690000	Suppurative laryngitis
ARI-Laryngitis	#85083002	Streptococcal laryngitis
ARI-Laryngitis	#8519009	Acute tracheitis with obstruction
ARI-Laryngitis	#85832003	Parainfluenza virus laryngitis
ARI-Laryngitis	#86773000	Oedematous laryngitis
ARI-Laryngitis	#95886009	Mycoplasmal tracheobronchitis
ARI-InfluenzaLikeIllness	#1033051000000101	Influenza due to zoonotic influenza virus
ARI-InfluenzaLikeIllness	#1033071000000105	Influenza due to pandemic influenza virus
ARI-InfluenzaLikeIllness	#1033091000000109	Influenza due to seasonal influenza virus
ARI-InfluenzaLikeIllness	#1033111000000104	Influenza with pneumonia due to seasonal influenza virus
ARI-InfluenzaLikeIllness	#10624911000119107	Otitis media due to H1N1 influenza
ARI-InfluenzaLikeIllness	#10624951000119108	Otitis media due to influenza
ARI-InfluenzaLikeIllness	#10628871000119101	Gastroenteritis due to influenza
ARI-InfluenzaLikeIllness	#10628911000119103	Gastroenteritis due to Influenza A virus
ARI-InfluenzaLikeIllness	#10629191000119100	Bronchiolitis caused by influenza virus
ARI-InfluenzaLikeIllness	#10629351000119108	Myocarditis due to Influenza A virus
ARI-InfluenzaLikeIllness	#10674911000119108	Otitis media due to Influenza A virus
ARI-InfluenzaLikeIllness	#10677711000119101	Encephalopathy due to Influenza A virus
ARI-InfluenzaLikeIllness	#10685111000119102	Upper respiratory tract infection due to Influenza
ARI-InfluenzaLikeIllness	#1149091008	Influenza caused by Influenza A virus subtype H2
ARI-InfluenzaLikeIllness	#142931000119100	Pneumonia due to H1N1 influenza
ARI-InfluenzaLikeIllness	#142941000119109	Upper respiratory tract infection due to H1N1 influenza
ARI-InfluenzaLikeIllness	#142951000119106	Myocarditis due to Influenza A virus subtype H1N1

ARI-InfluenzaLikeIllness	#142961000119108	Gastroenteritis due to H1N1 influenza
ARI-InfluenzaLikeIllness	#142971000119102	Encephalopathy due to H1N1 influenza
ARI-InfluenzaLikeIllness	#16311000119108	Pneumonia due to influenza
ARI-InfluenzaLikeIllness	#194946005	Acute myocarditis - influenzal
ARI-InfluenzaLikeIllness	#195878008	Pneumonia and influenza
ARI-InfluenzaLikeIllness	#195923003	Influenza with laryngitis
ARI-InfluenzaLikeIllness	#195924009	Influenza with pharyngitis
ARI-InfluenzaLikeIllness	#195929004	Influenza with gastrointestinal tract involvement
ARI-InfluenzaLikeIllness	#24662006	Influenza due to Influenza B virus
ARI-InfluenzaLikeIllness	#309789002	Encephalitis due to influenza
ARI-InfluenzaLikeIllness	#328531000119104	Upper respiratory tract infection due to Influenza A
ARI-InfluenzaLikeIllness	#41269000	Influenzal bronchopneumonia
ARI-InfluenzaLikeIllness	#43692000	Influenzal acute upper respiratory infection
ARI-InfluenzaLikeIllness	#442438000	Influenza due to Influenza A virus
ARI-InfluenzaLikeIllness	#442696006	Influenza due to Influenza A virus subtype H1N1
ARI-InfluenzaLikeIllness	#450715004	Influenza due to Influenza A virus subtype H7
ARI-InfluenzaLikeIllness	#450716003	Influenza due to Influenza A virus subtype H9
ARI-InfluenzaLikeIllness	#6142004	Influenza
ARI-InfluenzaLikeIllness	#61700007	Influenza with non-respiratory manifestation
ARI-InfluenzaLikeIllness	#707448003	Influenza due to Influenza A virus subtype H7N9
ARI-InfluenzaLikeIllness	#713083002	Influenza caused by Influenza A virus subtype H5
ARI-InfluenzaLikeIllness	#719590007	Influenza caused by seasonal influenza virus
ARI-InfluenzaLikeIllness	#719865001	Influenza caused by pandemic influenza virus
ARI-InfluenzaLikeIllness	#738276008	Influenza with CNS disorder
ARI-InfluenzaLikeIllness	#74644004	Influenza with encephalopathy
ARI-InfluenzaLikeIllness	#772810003	Influenza caused by Influenza A virus subtype H3N2
ARI-InfluenzaLikeIllness	#772828001	Influenza caused by Influenza A virus subtype H5N1
ARI-InfluenzaLikeIllness	#772839003	Pneumonia caused by Influenza A virus
ARI-InfluenzaLikeIllness	#78046005	Myocarditis due to influenza virus
ARI-InfluenzaLikeIllness	#81524006	Influenza due to Influenza C virus
ARI-InfluenzaLikeIllness	#866126000	Myelitis caused by Influenza A virus
ARI-InfluenzaLikeIllness	#95891005	Influenza-like illness
ARI-Croup	#22951000119104	Recurrent croup
ARI-Croup	#275495004	Acute fibrinous laryngotracheobronchitis
ARI-Croup	#35377009	Parainfluenza virus laryngotracheobronchitis
ARI-Croup	#71186008	Croup
ARI-Croup	#71255007	Adenoviral laryngotracheobronchitis

ARI-Croup	#72204002	Respiratory syncytial virus laryngotracheobronchitis
ARI-Croup	#73414003	Haemophilus influenzae laryngotracheobronchitis
ARI-Croup	#85915003	Laryngotracheobronchitis
ARI-Croup	#897121000000109	Westley Croup Score
ARI-Croup	#897141000000102	Assessment using Westley Croup Score
ARI-Croup	#897151000000104	Westley Croup Score
ARI-Pneumonia	#1010615002	Late syphilis of lung
ARI-Pneumonia	#1010620002	Infection of lung caused by Mycobacterium malmoense
ARI-Pneumonia	#1010622005	Infection of lung caused by Mycobacterium xenopi
ARI-Pneumonia	#1010634002	Pneumonia caused by Acinetobacter
ARI-Pneumonia	#1010662009	Infection of lung caused by Mycobacterium kansasii
ARI-Pneumonia	#1033111000000104	Influenza with pneumonia due to seasonal influenza virus
ARI-Pneumonia	#10624991000119103	Bronchopneumonia due to Achromobacter
ARI-Pneumonia	#10625031000119102	Bronchopneumonia due to anaerobic bacteria
ARI-Pneumonia	#10625071000119104	Bronchopneumonia due to bacteria
ARI-Pneumonia	#10625111000119106	Bronchopneumonia due to Escherichia coli
ARI-Pneumonia	#10625151000119107	Bronchopneumonia due to Group A Streptococcus
ARI-Pneumonia	#10625191000119102	Bronchopneumonia due to Group B Streptococcus
ARI-Pneumonia	#10625231000119106	Bronchopneumonia due to Haemophilus influenzae
ARI-Pneumonia	#10625271000119109	Bronchopneumonia due to Human metapneumovirus
ARI-Pneumonia	#10625311000119109	Bronchopneumonia due to Klebsiella pneumoniae
ARI-Pneumonia	#10625351000119105	Bronchopneumonia due to methicillin resistant Staphylococcus aureus
ARI-Pneumonia	#10625391000119100	Bronchopneumonia due to methicillin susceptible Staphylococcus aureus
ARI-Pneumonia	#10625431000119105	Bronchopneumonia due to Mycoplasma pneumoniae
ARI-Pneumonia	#10625471000119108	Bronchopneumonia due to Proteus mirabilis
ARI-Pneumonia	#10625511000119104	Bronchopneumonia due to Pseudomonas
ARI-Pneumonia	#10625551000119103	Bronchopneumonia due to respiratory syncytial virus
ARI-Pneumonia	#10625591000119108	Bronchopneumonia due to Staphylococcus
ARI-Pneumonia	#10625631000119108	Bronchopneumonia due to Staphylococcus aureus
ARI-Pneumonia	#10625671000119106	Bronchopneumonia due to Streptococcus
ARI-Pneumonia	#10625711000119105	Bronchopneumonia due to Streptococcus pneumoniae
ARI-Pneumonia	#10625751000119106	Bronchopneumonia due to virus
ARI-Pneumonia	#1087061000119106	Gonococcal pneumonia

ARI-Pneumonia	#1092361000119109	Rubella pneumonia
ARI-Pneumonia	#1092951000119106	Pneumonia due to Bordetella parapertussis
ARI-Pneumonia	#1149093006	Pneumonia caused by vancomycin resistant Enterococcus
ARI-Pneumonia	#1176988004	Enterobacter pneumonia
ARI-Pneumonia	#1187256004	Viral pneumonia due to Epstein-Barr virus infectious mononucleosis
ARI-Pneumonia	#120639003	Hantavirus pulmonary syndrome
ARI-Pneumonia	#1208602000	Pneumonia caused by Pseudomonas aeruginosa
ARI-Pneumonia	#123587001	Acute bronchopneumonia
ARI-Pneumonia	#123588006	Confluent bronchopneumonia with abscess formation
ARI-Pneumonia	#123589003	Necrotising bronchopneumonia
ARI-Pneumonia	#123590007	Focal pneumonia
ARI-Pneumonia	#123591006	Confluent pneumonia
ARI-Pneumonia	#124691000119101	Pneumonia due to methicillin resistant Staphylococcus aureus
ARI-Pneumonia	#12571000132104	Pneumonitis due to Herpes zoster
ARI-Pneumonia	#128711000119106	Pneumonia due to methicillin susceptible Staphylococcus aureus
ARI-Pneumonia	#142931000119100	Pneumonia due to H1N1 influenza
ARI-Pneumonia	#16311000119108	Pneumonia due to influenza
ARI-Pneumonia	#1731000119106	Atypical mycobacterial infection of lung
ARI-Pneumonia	#181007	Haemorrhagic bronchopneumonia
ARI-Pneumonia	#186342000	Pulmonary Mycobacterium avium complex infection
ARI-Pneumonia	#191727003	Post measles pneumonia
ARI-Pneumonia	#195878008	Pneumonia and influenza
ARI-Pneumonia	#195881003	Pneumonia due to respiratory syncytial virus
ARI-Pneumonia	#195886008	Group B streptococcal pneumonia
ARI-Pneumonia	#195888009	Proteus pneumonia
ARI-Pneumonia	#195889001	Legionella pneumonia
ARI-Pneumonia	#195896004	Pneumonia due to pleuropneumonia-like organism
ARI-Pneumonia	#195900001	Pneumonia due to measles
ARI-Pneumonia	#195902009	Anthrax pneumonia
ARI-Pneumonia	#195908008	Actinomycotic pneumonia
ARI-Pneumonia	#195909000	Nocardial pneumonia
ARI-Pneumonia	#195911009	Chickenpox pneumonia
ARI-Pneumonia	#196112005	Abscess of lung with pneumonia
ARI-Pneumonia	#2087000	Pulmonary nocardiosis
ARI-Pneumonia	#21846001	Pulmonary actinomycosis
ARI-Pneumonia	#22754005	Staphylococcal pneumonia
ARI-Pneumonia	#233604007	Pneumonia
ARI-Pneumonia	#233606009	Atypical pneumonia
ARI-Pneumonia	#233607000	Pneumococcal pneumonia
ARI-Pneumonia	#233608005	Meningococcal pneumonia

ARI-Pneumonia	#233609002	Chlamydial pneumonia
ARI-Pneumonia	#233617005	Haemorrhagic pneumonia
ARI-Pneumonia	#233618000	Mycobacterial pneumonia
ARI-Pneumonia	#233621003	Rickettsial pneumonia
ARI-Pneumonia	#233623000	Mononuclear interstitial pneumonia
ARI-Pneumonia	#233624006	Herpes simplex pneumonia
ARI-Pneumonia	#240387006	Pulmonary glanders
ARI-Pneumonia	#2523007	Salmonella pneumonia
ARI-Pneumonia	#266350000	Pneumococcal lobar pneumonia
ARI-Pneumonia	#277869007	Non-tuberculous mycobacterial pneumonia
ARI-Pneumonia	#278516003	Lobar pneumonia
ARI-Pneumonia	#300999006	Basal pneumonia
ARI-Pneumonia	#301000005	Left lower zone pneumonia
ARI-Pneumonia	#301001009	Right lower zone pneumonia
ARI-Pneumonia	#301002002	Left upper zone pneumonia
ARI-Pneumonia	#301003007	Right middle zone pneumonia
ARI-Pneumonia	#301004001	Right upper zone pneumonia
ARI-Pneumonia	#308906005	Secondary bacterial pneumonia
ARI-Pneumonia	#312342009	Infective pneumonia
ARI-Pneumonia	#31561003	Hypostatic bronchopneumonia
ARI-Pneumonia	#31920006	Haemorrhagic varicella pneumonitis
ARI-Pneumonia	#32204007	Pulmonary actinobacillosis
ARI-Pneumonia	#32286006	Pneumonia in Q fever
ARI-Pneumonia	#34020007	Pneumonia due to Streptococcus
ARI-Pneumonia	#35037009	Primary atypical interstitial pneumonia
ARI-Pneumonia	#35339003	Primary pneumonic plague
ARI-Pneumonia	#371072008	Postobstructive pneumonia
ARI-Pneumonia	#373435003	Battey disease
ARI-Pneumonia	#385093006	Community acquired pneumonia
ARI-Pneumonia	#38976008	Pneumonic plague
ARI-Pneumonia	#39172002	Pneumonia due to Proteus mirabilis
ARI-Pneumonia	#396285007	Bronchopneumonia
ARI-Pneumonia	#396286008	Bilateral bronchopneumonia
ARI-Pneumonia	#406595002	Infection due to Mycoplasma pneumoniae
ARI-Pneumonia	#407671000	Bilateral pneumonia
ARI-Pneumonia	#409664000	Pneumonia due to anaerobic bacteria
ARI-Pneumonia	#409665004	Pneumonia due to aerobic bacteria
ARI-Pneumonia	#41207000	Adenoviral pneumonia
ARI-Pneumonia	#41269000	Influenzal bronchopneumonia
ARI-Pneumonia	#41381004	Pneumonia due to Pseudomonas
ARI-Pneumonia	#420544002	Bacterial pneumonia with AIDS (acquired immunodeficiency syndrome)

ARI-Pneumonia	#420787001	Pneumococcal pneumonia with AIDS (acquired immunodeficiency syndrome)
ARI-Pneumonia	#421508002	Viral pneumonia with AIDS (acquired immunodeficiency syndrome)
ARI-Pneumonia	#421671002	Pneumonia with AIDS (acquired immunodeficiency syndrome)
ARI-Pneumonia	#425996009	Bilateral basal pneumonia
ARI-Pneumonia	#426696003	Lingular pneumonia
ARI-Pneumonia	#429271009	Ventilator associated pneumonia
ARI-Pneumonia	#430395005	Pneumonia due to Gram negative bacteria
ARI-Pneumonia	#440990006	Mycoplasma pneumoniae present
ARI-Pneumonia	#441590008	Pneumonia due to Severe acute respiratory syndrome coronavirus
ARI-Pneumonia	#441658007	Pneumonia due to Staphylococcus aureus
ARI-Pneumonia	#441942006	Pneumonia due to infection by Streptococcus pyogenes
ARI-Pneumonia	#445096001	Pneumonia due to Human metapneumovirus
ARI-Pneumonia	#446543007	Tuberculous abscess of lung
ARI-Pneumonia	#45312009	Pneumonia in typhoid fever
ARI-Pneumonia	#45556008	Pulmonary tularaemia
ARI-Pneumonia	#46970008	Mycoplasma pneumonia
ARI-Pneumonia	#471272001	Cavitary pneumonia
ARI-Pneumonia	#50804000	Catarrhal pneumonia
ARI-Pneumonia	#51530003	Pneumonia due to Escherichia coli
ARI-Pneumonia	#53084003	Bacterial pneumonia
ARI-Pneumonia	#55679008	Peribronchial pneumonia
ARI-Pneumonia	#57702005	Unresolved pneumonia
ARI-Pneumonia	#58890000	Adenoviral bronchopneumonia
ARI-Pneumonia	#59475000	Pneumonia in pertussis
ARI-Pneumonia	#60485005	Pleurobronchopneumonia
ARI-Pneumonia	#61884008	Achromobacter pneumonia
ARI-Pneumonia	#64479007	Pneumonia due to Klebsiella pneumoniae
ARI-Pneumonia	#64667001	Interstitial pneumonia
ARI-Pneumonia	#64703005	Terminal bronchopneumonia
ARI-Pneumonia	#64880000	Parainfluenza virus bronchopneumonia
ARI-Pneumonia	#64917006	Parainfluenza virus pneumonia
ARI-Pneumonia	#66429007	Unresolved lobar pneumonia
ARI-Pneumonia	#67525007	Secondary pneumonic plague
ARI-Pneumonia	#68409003	Organised pneumonia
ARI-Pneumonia	#70036007	Haemophilus influenzae pneumonia
ARI-Pneumonia	#7063008	Gangrenous pneumonia
ARI-Pneumonia	#713084008	Pneumonia caused by Human coronavirus
ARI-Pneumonia	#713544008	Bacterial pneumonia co-occurrent with human immunodeficiency virus infection

ARI-Pneumonia	#724498004	Pneumonia caused by Chlamydia pneumoniae
ARI-Pneumonia	#72656004	Granulomatous pneumonia
ARI-Pneumonia	#733051000	Pneumonia caused by Gram positive bacteria
ARI-Pneumonia	#75570004	Viral pneumonia
ARI-Pneumonia	#76090006	Pittsburgh pneumonia
ARI-Pneumonia	#763888005	Necrotising pneumonia caused by Panton-Valentine leukocidin producing Staphylococcus aureus
ARI-Pneumonia	#7678002	Cytomegaloviral pneumonia
ARI-Pneumonia	#772166001	Legionella pneumonia suspected
ARI-Pneumonia	#772839003	Pneumonia caused by Influenza A virus
ARI-Pneumonia	#81164001	Ornithosis with pneumonia
ARI-Pneumonia	#84353005	Pulmonary disease due to Mycobacteria
ARI-Pneumonia	#85469005	Hypostatic pneumonia
ARI-Pneumonia	#8555001	Syphilis of lung
ARI-Pneumonia	#882784691000119100	Pneumonia caused by SARS-CoV-2 (severe acute respiratory syndrome coronavirus 2)
ARI-Pneumonia	#95436008	Lung consolidation
ARI-Sinusitis	#111274000	Acute abscess of nasal sinus
ARI-Sinusitis	#11134001	Acute suppuration of sphenoidal sinus
ARI-Sinusitis	#13266007	Sphenoidal sinusitis
ARI-Sinusitis	#15805002	Acute sinusitis
ARI-Sinusitis	#16036000	Acute empyema of frontal sinus
ARI-Sinusitis	#17357005	Acute suppuration of frontal sinus
ARI-Sinusitis	#18643000	Ethmoidal sinusitis
ARI-Sinusitis	#195790000	Pansinusitis
ARI-Sinusitis	#232390009	Suppurative sinusitis with complications
ARI-Sinusitis	#23884004	Acute suppuration of maxillary sinus
ARI-Sinusitis	#25764005	Acute abscess of frontal sinus
ARI-Sinusitis	#27278006	Acute empyema of sphenoidal sinus
ARI-Sinusitis	#30239003	Acute abscess of sphenoidal sinus
ARI-Sinusitis	#35168006	Acute empyema of ethmoidal sinus
ARI-Sinusitis	#36971009	Sinusitis
ARI-Sinusitis	#371127003	Obstructive sinusitis
ARI-Sinusitis	#431231008	Acute rhinosinusitis
ARI-Sinusitis	#444814009	Viral sinusitis
ARI-Sinusitis	#5028002	Acute pansinusitis
ARI-Sinusitis	#58763001	Acute empyema of nasal sinus
ARI-Sinusitis	#61711004	Acute abscess of ethmoidal sinus
ARI-Sinusitis	#63140003	Acute suppuration of ethmoidal sinus
ARI-Sinusitis	#67832005	Acute ethmoidal sinusitis
ARI-Sinusitis	#68272006	Acute maxillary sinusitis
ARI-Sinusitis	#703470001	Bacterial sinusitis

ARI-Sinusitis	#721741006	Sinusitis caused by Streptococcus pneumoniae
ARI-Sinusitis	#721755003	Sinusitis caused by Haemophilus influenzae
ARI-Sinusitis	#725917007	Acute sinusitis caused by virus
ARI-Sinusitis	#75498004	Acute bacterial sinusitis
ARI-Sinusitis	#76653009	Acute empyema of maxillary sinus
ARI-Sinusitis	#77919000	Acute sphenoidal sinusitis
ARI-Sinusitis	#78737005	Frontal sinusitis
ARI-Sinusitis	#80600003	Acute suppuration of nasal sinus
ARI-Sinusitis	#88348008	Maxillary sinusitis
ARI-Sinusitis	#89194009	Acute abscess of maxillary sinus
ARI-Sinusitis	#897656009	Rhinosinusitis
ARI-Sinusitis	#91038008	Acute frontal sinusitis
ARI-TonsillitisPharyngitis	#10351008	Suppurative tonsillitis
ARI-TonsillitisPharyngitis	#111816002	Pneumococcal tonsillitis
ARI-TonsillitisPharyngitis	#11461005	Staphylococcal tonsillitis
ARI-TonsillitisPharyngitis	#126664009	Exudative pharyngitis
ARI-TonsillitisPharyngitis	#126665005	Oropharyngeal mucositis
ARI-TonsillitisPharyngitis	#14465002	Ulcerative tonsillitis
ARI-TonsillitisPharyngitis	#1532007	Viral pharyngitis
ARI-TonsillitisPharyngitis	#17741008	Acute tonsillitis
ARI-TonsillitisPharyngitis	#186659004	Herpangina
ARI-TonsillitisPharyngitis	#186675001	Viral pharyngoconjunctivitis
ARI-TonsillitisPharyngitis	#186963008	Vincent's angina
ARI-TonsillitisPharyngitis	#195655000	Acute gangrenous pharyngitis
ARI-TonsillitisPharyngitis	#195656004	Acute phlegmonous pharyngitis
ARI-TonsillitisPharyngitis	#195657008	Acute ulcerative pharyngitis
ARI-TonsillitisPharyngitis	#195658003	Acute bacterial pharyngitis
ARI-TonsillitisPharyngitis	#195659006	Acute pneumococcal pharyngitis
ARI-TonsillitisPharyngitis	#195660001	Acute staphylococcal pharyngitis
ARI-TonsillitisPharyngitis	#195662009	Acute viral pharyngitis
ARI-TonsillitisPharyngitis	#195666007	Acute erythematous tonsillitis
ARI-TonsillitisPharyngitis	#195667003	Acute follicular tonsillitis
ARI-TonsillitisPharyngitis	#195668008	Acute ulcerative tonsillitis
ARI-TonsillitisPharyngitis	#195669000	Acute catarrhal tonsillitis
ARI-TonsillitisPharyngitis	#195670004	Acute gangrenous tonsillitis
ARI-TonsillitisPharyngitis	#195671000	Acute bacterial tonsillitis
ARI-TonsillitisPharyngitis	#195672007	Acute pneumococcal tonsillitis
ARI-TonsillitisPharyngitis	#195673002	Acute staphylococcal tonsillitis
ARI-TonsillitisPharyngitis	#195676005	Acute viral tonsillitis
ARI-TonsillitisPharyngitis	#195709006	Pharyngolaryngitis
ARI-TonsillitisPharyngitis	#195804009	Lingular tonsillitis
ARI-TonsillitisPharyngitis	#195924009	Influenza with pharyngitis
ARI-TonsillitisPharyngitis	#232399005	Acute herpes simplex pharyngitis

ARI-TonsillitisPharyngitis	#232400003	Acute herpes zoster pharyngitis
ARI-TonsillitisPharyngitis	#232401004	Glandular fever pharyngitis
ARI-TonsillitisPharyngitis	#232402006	Meningococcal pharyngitis
ARI-TonsillitisPharyngitis	#232403001	Chlamydial pharyngitis
ARI-TonsillitisPharyngitis	#232417005	Vincent's tonsillitis
ARI-TonsillitisPharyngitis	#234528007	Nasopharyngeal sarcoidosis
ARI-TonsillitisPharyngitis	#240444009	Fusobacterial necrotising tonsillitis
ARI-TonsillitisPharyngitis	#240547000	Lymphonodular coxsackie pharyngitis
ARI-TonsillitisPharyngitis	#27878001	Follicular tonsillitis
ARI-TonsillitisPharyngitis	#302911003	Acute lingual tonsillitis
ARI-TonsillitisPharyngitis	#312422001	Infective pharyngitis
ARI-TonsillitisPharyngitis	#31309002	Respiratory syncytial virus pharyngitis
ARI-TonsillitisPharyngitis	#363746003	Acute pharyngitis
ARI-TonsillitisPharyngitis	#39271004	Ulcerative pharyngitis
ARI-TonsillitisPharyngitis	#405737000	Pharyngitis
ARI-TonsillitisPharyngitis	#40766000	Enteroviral lymphonodular pharyngitis
ARI-TonsillitisPharyngitis	#415724006	Tonsillitis due to Gram negative bacteria
ARI-TonsillitisPharyngitis	#41582007	Streptococcal tonsillitis
ARI-TonsillitisPharyngitis	#43878008	Streptococcal sore throat
ARI-TonsillitisPharyngitis	#51209006	Viral tonsillitis
ARI-TonsillitisPharyngitis	#51476001	Nasopharyngitis
ARI-TonsillitisPharyngitis	#55355000	Acute laryngopharyngitis
ARI-TonsillitisPharyngitis	#58031004	Suppurative pharyngitis
ARI-TonsillitisPharyngitis	#59221008	Parainfluenza virus rhinopharyngitis
ARI-TonsillitisPharyngitis	#59471009	Phlegmonous pharyngitis
ARI-TonsillitisPharyngitis	#652005	Gangrenous tonsillitis
ARI-TonsillitisPharyngitis	#703468005	Bacterial tonsillitis
ARI-TonsillitisPharyngitis	#70385007	Adenoviral pharyngoconjunctivitis
ARI-TonsillitisPharyngitis	#721586007	Pharyngotonsillitis caused by Human herpes simplex virus
ARI-TonsillitisPharyngitis	#72430001	Gangrenous pharyngitis
ARI-TonsillitisPharyngitis	#76651006	Pneumococcal pharyngitis
ARI-TonsillitisPharyngitis	#78430008	Adenoviral pharyngitis
ARI-TonsillitisPharyngitis	#78911000	Parainfluenza virus pharyngitis
ARI-TonsillitisPharyngitis	#82228008	Staphylococcal pharyngitis
ARI-TonsillitisPharyngitis	#878818001	Pharyngotonsillitis
ARI-TonsillitisPharyngitis	#90176007	Tonsillitis
ARI-TonsillitisPharyngitis	#95885008	Mycoplasmal pharyngitis
ARI-OtitisMedia	#10624911000119107	Otitis media due to H1N1 influenza
ARI-OtitisMedia	#10624951000119108	Otitis media due to influenza
ARI-OtitisMedia	#10674911000119108	Otitis media due to Influenza A virus
ARI-OtitisMedia	#1089341000119100	Otitis media of left ear
ARI-OtitisMedia	#1090001000119105	Otitis media due to scarlet fever
ARI-OtitisMedia	#1091951000119104	Otitis media of right ear

ARI-OtitisMedia	#1240521000000100	Otitis media due to disease caused by SARS-CoV-2 (severe acute respiratory syndrome coronavirus 2)
ARI-OtitisMedia	#14852000	Myringitis
ARI-OtitisMedia	#14948001	Acute suppurative otitis media without spontaneous rupture of ear drum
ARI-OtitisMedia	#16664009	Malignant otitis media
ARI-OtitisMedia	#19021002	Haemophilus influenzae otitis media
ARI-OtitisMedia	#19399000	Acute exudative otitis media
ARI-OtitisMedia	#194268005	Acute eustachian tube salpingitis
ARI-OtitisMedia	#194281003	Acute suppurative otitis media
ARI-OtitisMedia	#194286008	Bilateral suppurative otitis media
ARI-OtitisMedia	#194288009	Acute left otitis media
ARI-OtitisMedia	#194289001	Acute right otitis media
ARI-OtitisMedia	#194290005	Acute bilateral otitis media
ARI-OtitisMedia	#194311006	Acute myringitis without otitis media
ARI-OtitisMedia	#232245002	Granular myringitis
ARI-OtitisMedia	#270490007	Acute otitis media with effusion
ARI-OtitisMedia	#270491006	Eustachian tube salpingitis
ARI-OtitisMedia	#28795002	Subacute exudative otitis media
ARI-OtitisMedia	#29350000	Catarrhal otitis media
ARI-OtitisMedia	#297009	Acute myringitis
ARI-OtitisMedia	#3110003	Acute otitis media
ARI-OtitisMedia	#312218008	Infective otitis media
ARI-OtitisMedia	#32760002	Exudative otitis media
ARI-OtitisMedia	#33528003	Bullous myringitis
ARI-OtitisMedia	#360595002	Acute necrotising otitis media
ARI-OtitisMedia	#39288006	Purulent otitis media
ARI-OtitisMedia	#445172009	Cholesterol granuloma of middle ear and mastoid
ARI-OtitisMedia	#65363002	Otitis media
ARI-OtitisMedia	#703469002	Bacterial otitis media
ARI-OtitisMedia	#721742004	Otitis media caused by Streptococcus pneumoniae
ARI-OtitisMedia	#7271000119107	Acute bilateral otitis media with effusion
ARI-OtitisMedia	#7361000119105	Perforation of tympanic membrane due to otitis media
ARI-OtitisMedia	#84261000119106	Acute persistent otitis media
ARI-OtitisMedia	#85108007	Acute tubotympanic catarrh
ARI-OtitisMedia	#86279000	Acute suppurative otitis media with spontaneous rupture of ear drum
ARI-OtitisMedia	#89145009	Subacute tubotympanic catarrh
ARI-Bronchitis	#10509002	Acute bronchitis
ARI-Bronchitis	#111849006	Adenoviral bronchitis
ARI-Bronchitis	#1163489008	Human metapneumovirus bronchitis
ARI-Bronchitis	#13617004	Tracheobronchitis

ARI-Bronchitis	#138389411000119105	Acute bronchitis caused by SARS-CoV-2 (severe acute respiratory syndrome coronavirus 2)
ARI-Bronchitis	#16146001	Viral bronchitis
ARI-Bronchitis	#195714005	Acute fibrinous bronchitis
ARI-Bronchitis	#195717003	Acute purulent bronchitis
ARI-Bronchitis	#195719000	Acute pneumococcal bronchitis
ARI-Bronchitis	#195720006	Acute streptococcal bronchitis
ARI-Bronchitis	#195721005	Acute bronchitis caused by Haemophilus influenzae
ARI-Bronchitis	#195722003	Acute Moraxella catarrhalis bronchitis
ARI-Bronchitis	#195725001	Acute coxsackievirus bronchitis
ARI-Bronchitis	#195726000	Acute parainfluenza virus bronchitis
ARI-Bronchitis	#195727009	Acute respiratory syncytial virus bronchitis
ARI-Bronchitis	#195728004	Acute bronchitis due to rhinovirus
ARI-Bronchitis	#195729007	Acute echovirus bronchitis
ARI-Bronchitis	#233598009	Acute bacterial bronchitis
ARI-Bronchitis	#233599001	Acute mycoplasmal bronchitis
ARI-Bronchitis	#233600003	Acute chlamydial bronchitis
ARI-Bronchitis	#233601004	Acute viral bronchitis
ARI-Bronchitis	#233799004	Acute toxic tracheobronchitis
ARI-Bronchitis	#27475006	Parainfluenza virus bronchitis
ARI-Bronchitis	#29591002	Purulent bronchitis
ARI-Bronchitis	#312371005	Acute infective bronchitis
ARI-Bronchitis	#312400008	Acute infective tracheobronchitis
ARI-Bronchitis	#32398004	Bronchitis
ARI-Bronchitis	#35301006	Acute tracheobronchitis
ARI-Bronchitis	#405944004	Asthmatic bronchitis
ARI-Bronchitis	#40600002	Pneumococcal bronchitis
ARI-Bronchitis	#5875001	Acute bronchitis with obstruction
ARI-Bronchitis	#65878001	Septic bronchitis
ARI-Bronchitis	#714203003	Acute bronchitis co-occurrent with bronchiectasis
ARI-Bronchitis	#785728005	Bronchitis co-occurrent with wheeze
ARI-Bronchitis	#785737005	Bronchitis co-occurrent with chronic wheeze
ARI-Bronchitis	#785744001	Bronchitis co-occurrent with acute wheeze
ARI-Bronchitis	#785745000	Acute bronchitis co-occurrent with wheeze
ARI-Bronchitis	#79479005	Respiratory syncytial virus bronchitis
ARI-Bronchitis	#80257001	Acute bronchitis with bronchospasm
ARI-Bronchitis	#89549007	Catarrhal bronchitis
ARI-Bronchitis	#95886009	Mycoplasmal tracheobronchitis
ARI-URTI-NOS	#162388002	Has a sore throat
ARI-URTI-NOS	#162397003	Pain in throat
ARI-URTI-NOS	#267102003	Sore throat

ARI-URTI-NOS	#281794004	Viral upper respiratory tract infection
ARI-URTI-NOS	#54150009	Upper respiratory infection
ARI-URTI-NOS	#54398005	Acute upper respiratory infection
ARI-URTI-NOS	#82272006	Common cold
ARI-Bronchiolitis	#10629191000119100	Bronchiolitis caused by influenza virus
ARI-Bronchiolitis	#13089009	Adenoviral bronchiolitis
ARI-Bronchiolitis	#15199004	Acute bronchiolitis with bronchospasm
ARI-Bronchiolitis	#195737004	Acute exudative bronchiolitis
ARI-Bronchiolitis	#195739001	Acute bronchiolitis due to respiratory syncytial virus
ARI-Bronchiolitis	#233602006	Acute viral bronchiolitis
ARI-Bronchiolitis	#233603001	Acute bronchiolitis due to adenovirus
ARI-Bronchiolitis	#4120002	Bronchiolitis
ARI-Bronchiolitis	#445102008	Bronchiolitis due to Human metapneumovirus
ARI-Bronchiolitis	#52409006	Bronchiolitis exudativa
ARI-Bronchiolitis	#5505005	Acute bronchiolitis
ARI-Bronchiolitis	#57089007	Respiratory syncytial virus bronchiolitis
ARI-Bronchiolitis	#718004	Acute bronchiolitis with obstruction
ARI-Bronchiolitis	#87695000	Necrotising bronchiolitis
ARI-LRTI-NOS	#128601007	Infectious disease of lung
ARI-LRTI-NOS	#161924005	Productive cough -green sputum
ARI-LRTI-NOS	#161925006	Productive cough-yellow sputum
ARI-LRTI-NOS	#162951005	O/E - bronchial breathing
ARI-LRTI-NOS	#195742007	Acute lower respiratory tract infection
ARI-LRTI-NOS	#24816000	Rusty sputum
ARI-LRTI-NOS	#248599002	Copious sputum
ARI-LRTI-NOS	#248600004	Moderate sputum
ARI-LRTI-NOS	#248605009	Dirty sputum
ARI-LRTI-NOS	#271827002	Sputum abnormal - colour
ARI-LRTI-NOS	#274400006	Pus in sputum O/E
ARI-LRTI-NOS	#275497007	Infection of lower respiratory tract and mediastinum
ARI-LRTI-NOS	#277900008	Grey sputum
ARI-LRTI-NOS	#277903005	Thick sputum
ARI-LRTI-NOS	#277907006	Yellow sputum
ARI-LRTI-NOS	#277908001	Green sputum
ARI-LRTI-NOS	#277910004	Brown sputum
ARI-LRTI-NOS	#28743005	Productive cough
ARI-LRTI-NOS	#312119006	Bacterial lower respiratory infection
ARI-LRTI-NOS	#312134000	Viral lower respiratory infection
ARI-LRTI-NOS	#42192008	Purulent sputum
ARI-LRTI-NOS	#50417007	Lower respiratory tract infection
ARI-LRTI-NOS	#79451004	Bronchial breathing
ARI-LRTI-NOS	#860741000000100	Dark green sputum
ARI-LRTI-NOS	#860761000000104	Pale green sputum

ARI-LRTI-NOS	#880529761000119102	Lower respiratory infection caused by SARS-CoV-2 (severe acute respiratory syndrome coronavirus 2)
ARI-LRTI-NOS	#8955008	Mucopurulent sputum
ARI-AsthmaExacerbation	#10674711000119105	Acute severe exacerbation of asthma co-occurrent with allergic rhinitis
ARI-AsthmaExacerbation	#10674791000119101	Acute exacerbation of intermittent allergic asthma
ARI-AsthmaExacerbation	#10675471000119109	Acute severe exacerbation of severe persistent allergic asthma
ARI-AsthmaExacerbation	#10675551000119104	Acute severe exacerbation of severe persistent asthma co-occurrent with allergic rhinitis
ARI-AsthmaExacerbation	#10675911000119109	Acute severe exacerbation of mild persistent allergic asthma
ARI-AsthmaExacerbation	#10675991000119100	Acute severe exacerbation of mild persistent allergic asthma co-occurrent with allergic rhinitis
ARI-AsthmaExacerbation	#10676271000119104	Acute exacerbation of moderate persistent allergic asthma
ARI-AsthmaExacerbation	#10676431000119103	Acute severe exacerbation of moderate persistent allergic asthma
ARI-AsthmaExacerbation	#10676511000119109	Acute severe exacerbation of moderate persistent asthma co-occurrent with allergic rhinitis
ARI-AsthmaExacerbation	#10692721000119102	Chronic obstructive asthma co-occurrent with acute exacerbation of asthma
ARI-AsthmaExacerbation	#1086701000000102	Life threatening acute exacerbation of allergic asthma
ARI-AsthmaExacerbation	#1086711000000100	Life threatening acute exacerbation of intrinsic asthma
ARI-AsthmaExacerbation	#1751000119100	Acute exacerbation of chronic obstructive airways disease with asthma
ARI-AsthmaExacerbation	#281239006	Acute asthma
ARI-AsthmaExacerbation	#425969006	Exacerbation of intermittent asthma
ARI-AsthmaExacerbation	#442025000	Acute exacerbation of chronic asthmatic bronchitis
ARI-AsthmaExacerbation	#707445000	Exacerbation of mild persistent asthma
ARI-AsthmaExacerbation	#707446004	Exacerbation of moderate persistent asthma
ARI-AsthmaExacerbation	#707447008	Exacerbation of severe persistent asthma
ARI-AsthmaExacerbation	#707979007	Acute severe exacerbation of severe persistent asthma
ARI-AsthmaExacerbation	#707980005	Acute severe exacerbation of moderate persistent asthma
ARI-AsthmaExacerbation	#707981009	Acute severe exacerbation of mild persistent asthma
ARI-AsthmaExacerbation	#708090002	Acute severe exacerbation of asthma
ARI-AsthmaExacerbation	#708093000	Acute exacerbation of allergic asthma
ARI-AsthmaExacerbation	#708094006	Acute exacerbation of intrinsic asthma
ARI-AsthmaExacerbation	#708095007	Acute severe exacerbation of immunoglobin E-mediated allergic asthma
ARI-AsthmaExacerbation	#708096008	Acute severe exacerbation of intrinsic asthma

ARI-AsthmaExacerbation	#733858005	Acute severe refractory exacerbation of asthma
ARI-AsthmaExacerbation	#734904007	Life threatening acute exacerbation of asthma
ARI-AsthmaExacerbation	#734905008	Moderate acute exacerbation of asthma
ARI-AsthmaExacerbation	#762521001	Exacerbation of allergic asthma
ARI-AsthmaExacerbation	#782513000	Acute severe exacerbation of allergic asthma
ARI-AsthmaExacerbation	#782520007	Exacerbation of allergic asthma due to infection
ARI-AsthmaExacerbation	#786836003	Near fatal asthma
ARI-AsthmaExacerbation	#829976001	Thunderstorm asthma
ARI-AsthmaExacerbation	#99031000119107	Acute exacerbation of asthma co-occurrent with allergic rhinitis
ARI-COPDExacerbation	#106001000119101	Chronic obstructive lung disease co-occurrent with acute bronchitis
ARI-COPDExacerbation	#1751000119100	Acute exacerbation of chronic obstructive airways disease with asthma
ARI-COPDExacerbation	#195951007	Acute exacerbation of chronic obstructive airways disease
ARI-COPDExacerbation	#196001008	Chronic obstructive pulmonary disease with acute lower respiratory infection
ARI-COPDExacerbation	#285381006	Acute infective exacerbation of chronic obstructive airways disease
ARI-COPDExacerbation	#293241000119100	Acute exacerbation of chronic obstructive bronchitis
ARI-COPDExacerbation	#408501008	Emergency hospital admission for chronic obstructive pulmonary disease
ARI-COPDExacerbation	#425748003	Acute exacerbation of chronic bronchitis
ARI-COPDExacerbation	#847091000000104	Acute non-infective exacerbation of chronic obstructive pulmonary disease
ARI-ExacerOther	#445378003	Acute exacerbation of bronchiectasis
ARI-ExacerOther	#525691000000103	Antibiotic therapy for acute pulmonary exacerbation
ARI-ExacerOther	#789574002	Acute exacerbation of idiopathic pulmonary fibrosis
ARI-ExacerOther	#789574002	Acute exacerbation of idiopathic pulmonary fibrosis
ARI-ExacerOther	#859041000000103	Exacerbation of cystic fibrosis
ARI-ExacerOther	#879963005	Exacerbation of bronchiectasis caused by infection
ARI-ExacerOther	#12591000132100	Suspected SARS (severe acute respiratory syndrome)
ARI-ExacerOther	#135883003	Cough with fever
ARI-ExacerOther	#195647007	Acute respiratory infections
ARI-ExacerOther	#275498002	Respiratory tract infection
ARI-ExacerOther	#312133006	Viral respiratory infection
ARI-ExacerOther	#707224005	Severe acute respiratory infection
ARI-CovidSuspected	#1017214008	SARS-CoV-2 viraemia
ARI-CovidSuspected	#1119302008	Acute COVID-19
ARI-CovidSuspected	#1240451000000106	Telephone consultation for suspected SARS-CoV-2 (severe acute respiratory syndrome coronavirus 2)

ARI-CovidSuspected	#1300731000000106	COVID-19 confirmed using clinical diagnostic criteria
ARI-CovidSuspected	#1325171000000109	Acute COVID-19 infection
ARI-CovidSuspected	#700217006	Suspected coronavirus infection
ARI-CovidSuspected	#840539006	COVID-19
ARI-CovidSuspected	#840544004	Suspected COVID-19

SUPPLEMENT S6: DATA SECUITY PROCEDURES AT THE RSC

Infrastructure: ORCHID secure servers are held physically in a locked and monitored data centre in Oxford with highly restricted access. Access, management and analysis of patient level pseudonymised data is only possible within restricted folders of the secure ORCHID server via a virtual private network (VPN) and requires multifactor authentication (MFA). Transfer of data outside of the secure server follows a strict approval process. Only aggregate and summary data can be transferred outside the secure server. Individuals accessing the data need to undertake appropriate annual data security training.

Statistical disclosure controls (SDCs): These are methods designed to protect the confidentiality of individuals' data in this study while still allowing for the publication of useful and accurate statistics.

1. **Anonymisation:** Although the data is not totally anonymised, all directly identifiable personal data is removed; no names or contact details are included.
2. **Pseudonymisation:** The NHS number is pseudonymised using a hashing algorithm (prior to receipt of the data). This converts the NHS number to a fixed number of digits totally distinct from the original NHS number. Although hashing is a one-way process if an individual has knowledge of an NHS number and its equivalent hashed version they could, in theory, identify the algorithm. To counter this, what's known as a salt is used. This is where a random sequence is added to the NHS numbers before hashing occurs. Without access to the salt it would be essentially impossible to determine the NHS numbers from their hashed equivalent. Data can only be formally linked back to other data sets with NHS numbers if exactly the same algorithm is applied to the data with the same salt.
3. **Perturbation:** Dates of birth have been rounded to the nearest month.
4. **Aggregation:** Data transferred out of the secure server will be summary statistics only. Thus will no longer be regarded as personal data.

Data minimisation: Only data required for the purpose of the study will be held within ORCHID folder within the secure server. As a consequence of these measures, identification of individuals directly or indirectly using linked data is highly unlikely, even by research members, as the data used in this specific project will be limited to that which is essential to fulfil the objectives. Identifying the individuals by triangulation of these limited data points would be exceptionally unlikely.

SUPPLEMENT S7: VENN DIAGRAM OF CODLEIST

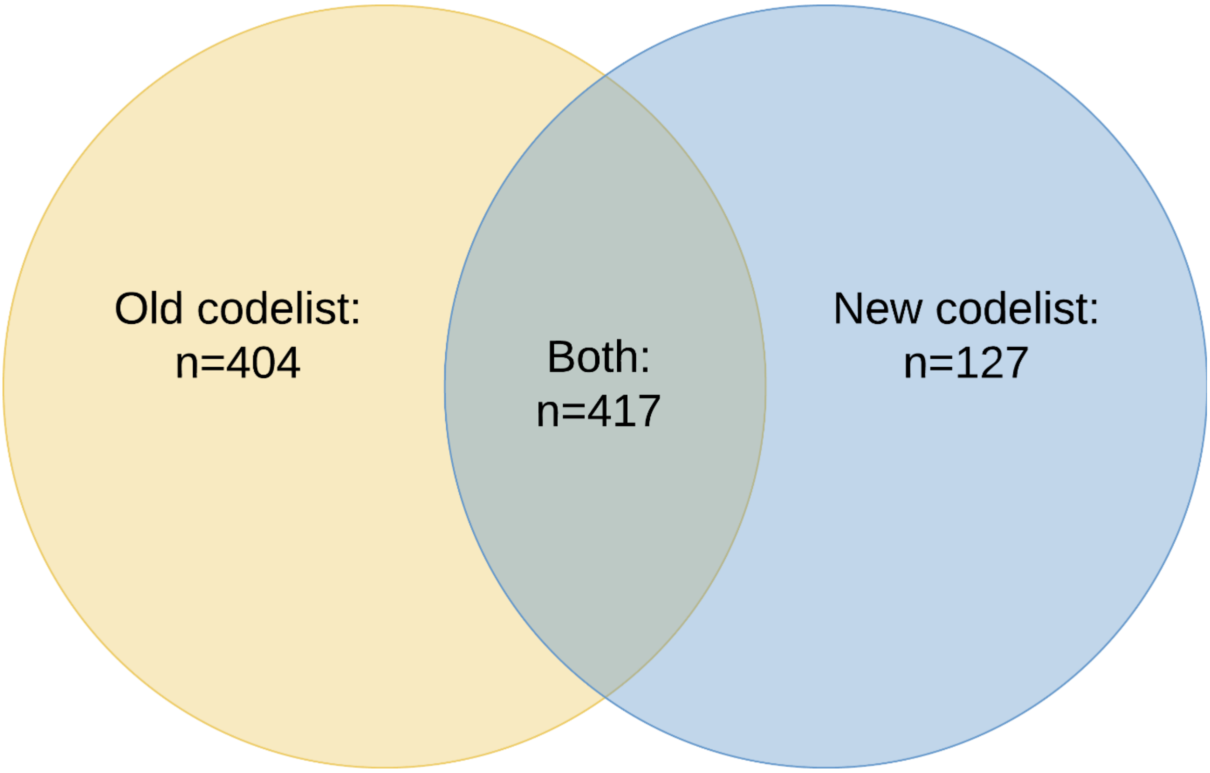

The old codelists included 404+417=821 codes and the new codelist included 127+417=544 codes. 417 codes appeared in both codelists, 404 only in the old and 127 only in the new codelists.

SUPPLEMENT S8: ARI INCIDENCE BY AGE AND RISK GROUP

			Total case during study			Weekly rate / 100k	
Indicator	Risk group	Age band	New	Old	% Change	New	Old
ARI	Non-risk	0-17	813,364	710,060	14.55	436.50	375.95
		18-69	726,490	565,643	28.44	148.80	115.15
		70+	51,965	41,039	26.62	128.05	101.00
	Risk	0-17	91,521	71,978	27.15	480.45	387.90
		18-69	513,302	363,681	41.14	336.50	234.85
		70+	281,831	212,940	32.35	339.55	254.15
ILI	Non-risk	0-17	6,879	6,879	0.00	1.95	1.95
		18-69	22,233	22,234	0.00	3.00	3.00
		70+	1,039	1,039	0.00	1.50	1.50
	Risk	0-17	1,006	1,006	0.00	2.60	2.60
		18-69	10,766	10,767	-0.01	4.55	4.55
		70+	4,603	4,604	-0.02	2.80	2.80
LRTI	Non-risk	0-17	116,620	108,154	7.83	56.55	54.70
		18-69	223,505	150,058	48.95	44.80	30.05
		70+	31,293	22,894	36.69	76.35	54.25
	Risk	0-17	17,512	14,333	22.18	89.30	74.30
		18-69	244,124	179,728	35.83	159.35	116.40
		70+	194,010	163,895	18.37	232.80	197.65
URTI	Non-risk	0-17	707,263	634,517	11.46	381.45	337.70
		18-69	490,165	408,016	20.13	98.80	82.30
		70+	19,746	18,322	7.77	50.25	46.60
	Risk	0-17	70,250	60,735	15.67	369.70	327.10
		18-69	215,760	185,560	16.28	137.10	118.10
		70+	53,697	50,782	5.74	63.40	57.30

S7: Median weekly rate for level-1 and level-2 Acute Respiratory infection (ARI) indicators by age band and risk group compared to the previous indicators. The rate is based on denominator of 17,433,074 subjects. % change = percent change in total cases over the study period. ILI: influenza-like illness, ECLD: exacerbations of chronic lung disease. LRTI: lower respiratory tract infection, URTI: upper respiratory tract infection. Note that ECLD is a new indicator and does not have a suitable comparator. The totals numerators of level-2 indicators do not add up to the ARI numerator as we have not included cases from the level-2 ARI-NOS category.
